# Contraceptive Options and Their Associated Estrogenic Environmental Loads: Relationships and Trade-Offs

**DOI:** 10.1371/journal.pone.0092630

**Published:** 2014-03-26

**Authors:** Usman Khan, Jim A. Nicell

**Affiliations:** Department of Civil Engineering & Applied Mechanics, McGill University, Montreal, Quebec, Canada; China Agricultural University, China

## Abstract

This work explores the relationships between a user's choice of a given contraceptive option and the load of steroidal estrogens that can be associated with that choice. Family planning data for the USA served as a basis for the analysis. The results showed that collectively the use of contraception in the USA conservatively averts the release of approximately 4.8 tonnes of estradiol equivalents to the environment. 35% of the estrogenic load released over the course of all experienced pregnancies events and 34% the estrogenic load represented by all resultant legacies are a result of contraception failure and the non-use of contraception. A scenario analysis conducted to explore the impacts of discontinuing the use of ethinylestradiol-based oral contraceptives revealed that this would not only result in a 1.7-fold increase in the estrogenic loading of the users, but the users would also be expected to experience undesired family planning outcomes at a rate that is 3.3 times higher. Additional scenario analyses in which ethinylestradiol-based oral contraceptive users were modeled as having switched entirely to the use of male condoms, diaphragms or copper IUDs suggested that whether a higher or lower estrogenic load can be associated with the switching population depends on the typical failure rates of the options adopted following discontinuation. And, finally, it was estimated that, in the USA, at most 13% of the annual estrogenic load can be averted by fully meeting the contraceptive needs of the population. Therefore, while the issue of estrogen impacts on the environment cannot be addressed solely by meeting the population's contraceptive needs, a significant fraction of the estrogenic mass released to environment can be averted by improving the level with which their contraceptive needs are met.

## Introduction

The environmental release of natural and synthetic steroidal estrogens is of concern because it is suspected that these compounds are major causative agents of fish feminization and other associated environmental impacts [1]–[10]. Consequently, ethinylestradiol (EE_2_), the synthetic estrogen used in birth control pills, and estradiol (E_2_), the most potent natural estrogen, are being considered for regulation by the European Union with proposed Environmental Quality Standards of 35 and 400 pg/L, respectively [11], [12]. Proposed Swiss standards are very similar [13]. However, an industry led effort [14] proposed no-effect benchmarks for these estrogens at levels that are significantly higher than those proposed by the European Union and Switzerland.

EE_2_ is primarily released due to its use in predominant oral contraceptives and, more recently, due to its use in transdermal patches and vaginal rings [15]. In addition to being endogenously produced, E_2_ is released into the environment through the use of hormone replacement therapy (HRT) preparations and recently due to the use of Natazia, a one-of-a-kind combined oral contraceptive (OC) pill containing E_2_ instead of EE_2_ as the estrogen [15]–[21]. Two other steroidal estrogens, namely estrone (E_1_) and estriol (E_3_), have also recently drawn regulatory interest [22]. In addition to being endogenously produced, E_1_ and E_3_ are released into the environment due to their use in various HRT preparations [16]–[19].

**Figure 1:**
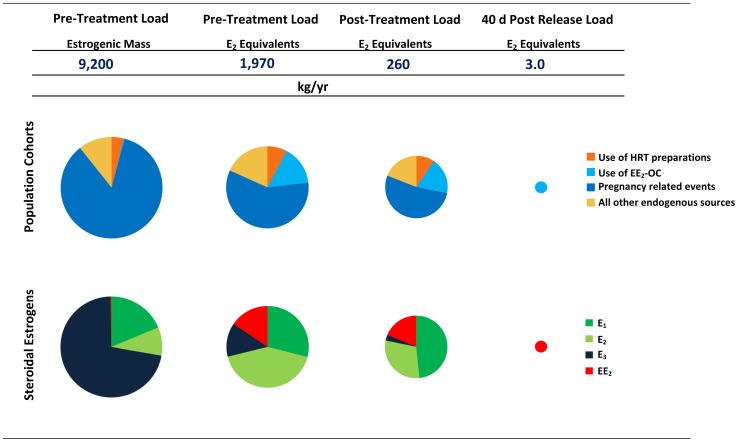
Estimated Steroidal Estrogen Loads in the USA circa 2002. The relative size of the pie charts is proportional to the logarithm of the estimated loads. Estrogen masses were estimated using the data compiled in File S1. Estradiol (E_2_) equivalents were estimated by summing the respective mass loads of each estrogen, weighted according to their estrogenic potencies relative to estradiol, as follows: [E_1_]/3+[E_2_]+[E_3_]/25+10·[EE_2_]. The justifications for potencies weightings used in this equation are detailed in File S2.

Approximately 1,970 kg of estrogens, expressed as estradiol equivalents (E_2-eq_), are released each year to sewage treatment plants in the United States of America (USA) for treatment (Figure 1). Of this mass, after undergoing wastewater treatment, an estimated 260 kg of E_2-eq_ are discharged to waterways in the USA. Forty days after release, which is a typical residence time of a wastewater parcel in rivers [23], only 3 kg of the original discharged E_2-eq_ load are expected to remain (Figure 1). Of particular note is the fact that the release of natural estrogens due to all pregnancy-related events accounts for 59% of the post-treatment load and an additional 16% of this load arises due to the direct release of EE_2_ from the use of oral contraceptives (Figure 1). However, EE_2_ is considerably more persistent than E_1_, E_2_ and E_3_ [17], [24], [25] and, hence, even though the net E_2-eq_ river laden load will decrease over time, the fraction of that load that is due to residual presence of EE_2_ will steadily increase from 16%. For example, consider that after 40 days, it is estimated that only about 1% of the initially released load would remain, but almost 100% of this load would be due to the residual presence of EE_2_ (Figure 1). Hence, the loads that arise from pregnancy-related events and those released due to the use of EE_2_ are important.

To-date, estrogen loads arising from all pregnancy-related events and those arising from the release of EE_2_ due its use in oral contraceptives have been treated as mutually independent in literature [16], [26]–[28]. We contend that this is problematic since it leads to the misconception that the use of an ethinylestradiol-based oral contraceptive (EE_2_-OC) is the only form of contraception that has an estrogenic load associated with its use and, by extension of this, other forms of contraception are presumed to not have any associated estrogenic loads. This misinterpretation largely arises from the belief that the use of a particular contraceptive option only results in an estrogenic load if the option itself is estrogen-based [26], [27]; however, this is not entirely true given that every contraceptive option fails to some extent [29]. Such failures lead to unintended pregnancies, the result of which is a temporary increase in the excretion of natural steroidal estrogens over the course of the pregnancy. Hence, at the very least, the choice to use each contraceptive option has a load of natural steroidal estrogens associated with its use. Therefore, it can be said that a fraction of the overall pregnancy load, which by far is the single most important contributor to the net estrogenic load (Figure 1), results from the failure of contraceptive choices made by the population. Consider that nearly half of the pregnancy events experienced in the USA are unintended [30], [31]. However, the fraction of the estrogenic load released over the course of all pregnancy events that are unintended (and, hence, are due to the failure and non-use of methods of contraception) remains to be quantified.

The recognition that each contraceptive option has an estrogenic load of natural hormones associated with its use leads to an important question: that is, how do the estrogenic loads associated with various contraceptive options compare? For example, consider the work of Wise et al. [26] who suggested that one way of reducing the EE_2_ load on the environment would be for some EE_2_-OC users to switch to non-hormonal methods of contraception such as copper intrauterine devices (copper IUD), diaphragms, or male condoms. As much as this suggestion holds true when considering the direct release of EE_2_ alone, the recognition that each contraceptive option has an indirect estrogenic load associated with it use (i.e., due to the failure of the option) requires that suggestions such as those of Wise et al.'s be revaluated with a renewed focus. That is, it should be asked how the total environmental load of steroidal estrogens would likely change if a given group of users switched from using EE_2_-OC to such alternative methods. Moreover, the change in the overall load of steroidal estrogens should be estimated for those EE_2_-OC users who discontinue the use of their current contraceptive option by switching to other methods or by abandoning the use of contraception altogether (Note: currently, one-third of the EE_2_-OC users discontinue the use of their option within the first year [29]). The objective of such an evaluation would be to assess the change in the associated estrogenic loading of an EE_2_-user when she chooses to discontinue the use of her current option. The impacts of such decisions on estrogenic loading of an EE_2_-user have yet to be conceptually recognized or mathematically modeled in literature.

Furthermore, not only does the use of each contraceptive option have an estrogenic load associated with its use, but the use of each also prevents an estrogenic load from being released to the environment. That is, given that the use of every contraceptive option, when compared to not using any method at all, averts a number of pregnancies [29], this prevents a load of natural estrogens from being released to the environment from the averted pregnancies and by subsequent generations of offspring. Therefore, it can be qualitatively stated that a population's use of each non-estrogen based contraceptive method on a net basis prevents the release of an estrogenic load to the environment. Such an assertion is not directly applicable for those contraceptive options that are themselves estrogen-based since the use of such estrogens-based options invariably involves estrogenic loading trade-offs. Consider that, on the one hand, the use of such options averts an estrogenic load from being released through the prevention of pregnancies and, on the other hand, their use leads to the direct release of estrogenic load via the excreta of respective users. Such considerations have largely remained unacknowledged in the literature to-date and, hence, no estimates, or models to arrive at them, are currently available to quantify the total estrogenic load that is averted through a population's collective use of contraception or specifically averted through the use of each given option.

The above discussion highlights why it is necessary that the relationships between the choice of using a given contraceptive option and the associated estrogenic load should be examined in more than a cursory fashion. This is especially important given that the regulation of steroidal estrogens, and most particularly that of EE_2_, is likely to engender considerable public debate [27], [32]–[35], [65] with important implications for both the environment and human reproductive health. Thus, a better understanding of the relationships between contraceptive options and their associated loads of steroidal estrogens on the environment is required in order to fully inform this debate. This is the overall objective of the present study. Note that, in order to put the importance of the estrogen load for each contraceptive option into a proper perspective, other considerations with respect to parental planning and public health implications will be briefly discussed, where relevant.

## Methods and Models

Unless otherwise indicated, all estrogenic loads discussed below are calculated on a pre-treatment basis; i.e., loads discharged by a population into the sewer system prior to their treatment and/or release into the environment. While the models used in this study were developed to be universally applicable, the parameterization of the models and their application were performed using data from the USA, for which extensive data sets were available. The reference year for most data is circa 2002.

### 1. Estrogenic Equivalents

Since estrogens act in an additive manner [36] and since the eco-toxicological potency of all estrogens is not equal [11], [12], [17], [18], [24], [36] there is a need to express the mass loads of the various estrogens on an equivalence basis. In the present study, the net estrogenic loads are expressed as equivalents of estradiol (E_2-eq_), the most potent natural estrogen. Specifically, for this evaluation, EE_2_, E_1_ and E_3_ were assumed to be 10 [11], [12], 0.33 [24] and 0.04 [37], [38] times as potent as E_2_, respectively. For the rationale behind the selected potencies, see File S2.

### 2. Net Estrogen Load Due to a Population's Use of a Given Contraceptive Option

**Figure 2:**
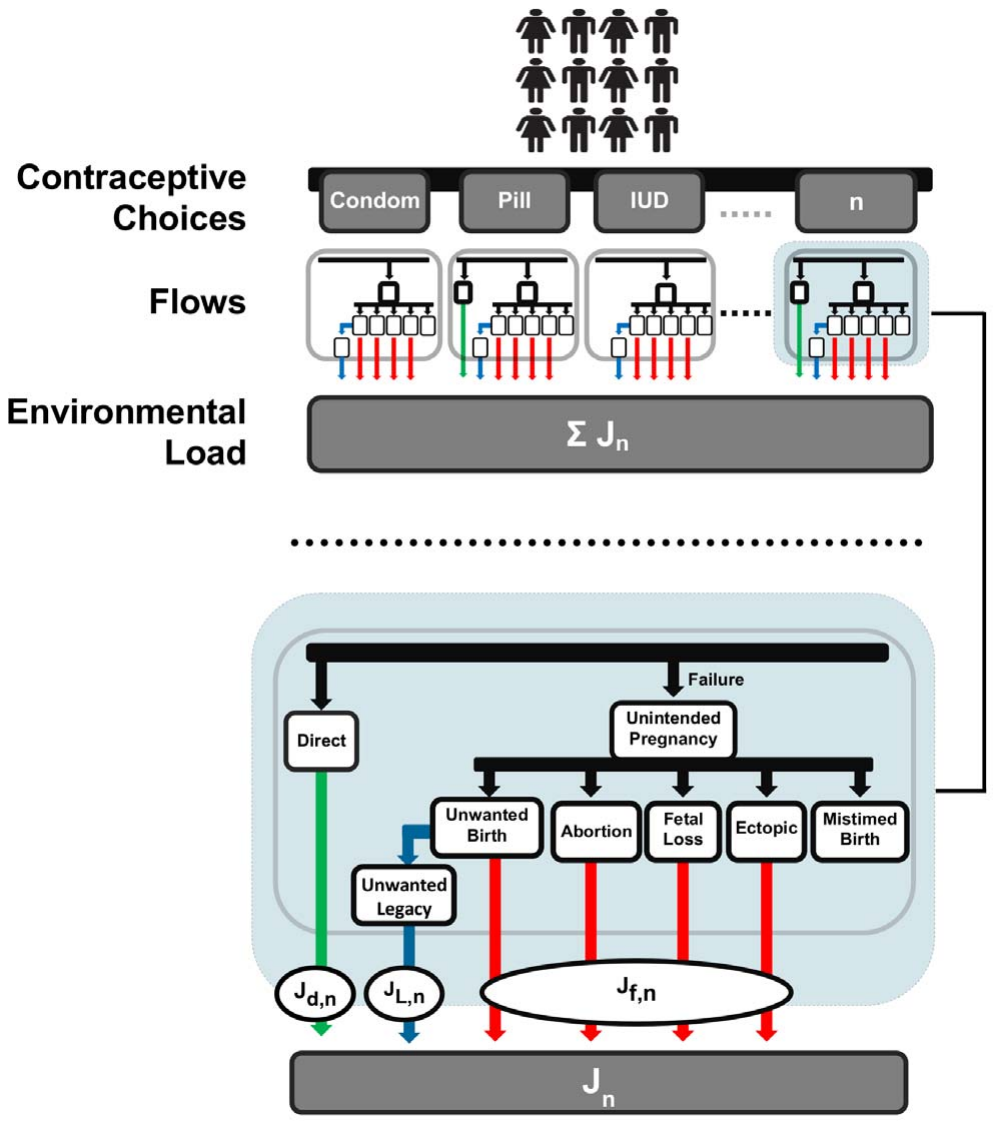
Relationship between contraceptive choices and the resultant flows of steroidal estrogens (i.e., through direct excretion and contraceptive failure) contributing to the net load of steroidal estrogens attributed to the use of a particular contraceptive option (J_n_) or the total of all options (i.e., ΣJ_n_). Note that since mistimed births only lead to time-displaced estrogenic flows such pregnancies are not identified as a source of steroidal estrogenic attributable to a user's choice of a particular contraceptive option.

The objective of this research is to examine the implications of the use of various forms of contraception by accounting for all estrogenic loads attributable to the use of a given contraceptive option. Conceptually, the net environmental estrogenic load associated with the use of a given option, *n*, is composed of three distinct contributions represented by the variables J*_d,n_*, J*_f,n_,* and J*_L,n_* (see Figure 2).

The most obvious of these and the only one directly acknowledged in the literature to-date is J*_d,n_*, which is the contribution that is directly released upon use of a contraceptive option, *n*, via the excreta of respective users. This contribution is non-zero for estrogen-based contraceptive options only. More specifically, of the various contraceptive options considered here, the contribution *J_d,n_* is only relevant for EE_2_-based preparations (EE_2_-OC) and the recently authorized E_2_-based oral contraceptives (E_2_-OC). See File S3 for a detailed discussion on the modelling of this contribution and the parameterization of the resulting model.

An additional contribution, J*_f,n_*, arises from the recognition that all contraceptive options will occasionally fail (see Figure 2). Failure of a contraceptive option will often lead to an *unintended pregnancy*, which refers to a pregnancy that is undesired at the time of conception and occurs either through the failure of the contraceptive option being used or the non-use of contraception altogether [29]. A pregnancy leads to a significant increase in the endogenous excretion of E_1_, E_2_ and E_3_. The magnitude of this increase is a function of the duration of the pregnancy [39], [40], which is directly related to its outcome (i.e., birth, induced abortion, spontaneous abortion, and ectopic pregnancy) [41]. It is important to note, however, that the load of natural estrogens released over the course of an unintended pregnancy resulting in the outcome of birth, should not be fully attributed to the parents' choice of using a given contraceptive option. That is, consider that births that result from unintended pregnancies are classified in family planning literature as either being *mistimed* or *unwanted* [42]–[44]. A *mistimed* birth is a time-displaced birth in that it occurs earlier than desired [44]. Hence, the estrogenic load associated with such a birth can be considered to be simply displaced in time and, therefore, should not be attributed to the parents' choice of a given contraceptive option. In contrast, an *unwanted* birth can be viewed as an “extra” birth, since it occurs despite the couple's lack of intent at the time of conception to have a child in the future [44]. Since *unwanted* births are “extra” births that occur because the needs of the parents could not be fully met by their contraceptive method(s) of choice, the estrogenic load released over the course of the resultant unintended pregnancies should be attributed to the parents' contraceptive choices. This consideration does not apply to all other outcomes of unintended pregnancies (i.e., induced abortion, spontaneous abortion and ectopic) since these outcomes do not end up satisfying a future need of the parents for a child. See File S4 for a detailed discussion on how the estrogen contributions arising from unintended pregnancies were modeled and how the resulting model was parameterized.

In addition to leading to the release of natural estrogens over the course of the resultant unintended pregnancy, the failure of a contraceptive option is also a source of an additional contribution, J*_L,n_*, due to unintended pregnancies that ultimately lead to the outcome of *unwanted* births (Figure 2). Since *unwanted* births are “extra” births that occur because the family planning expectations of the parents were not fully met by their contraceptive method, the estrogenic load released over the course of the unwanted child's lifespan as well that person's genetic lineage may also be attributed to the parents' choice to use a given contraceptive option. In this way, such a contribution can be viewed as a “legacy” load since it will manifest while the genetic lineage of the born unwanted child remains alive. See File S5 for details on how this estrogen contribution was modeled and how the resulting model was parameterized.

Overall, the estrogenic load that can be associated with the choice to use a given contraception *n* by a given user can be estimated by summing the respective contributions J*_d,n_*, J*_f,n_* and J*_L,n_* as follows (see Figure 2): 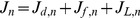
(1)


Eq. (1) forms the basis for the evaluation of all contraceptive methods and scenarios presented below.

### 3. Data and Models

As will be presented below, extensive analyses were conducted to understand how the choice to use a given contraceptive option or contraception collectively relates to the anthropogenic load of steroidal estrogens released to the environment.

The Supporting Information sections include details on the development of models that were applied in this study to estimate the:

- estrogen load released directly via the use of a particular contraceptive option, J*_d,n_* (see File S3);
- natural estrogen load released over the course of unintended pregnancies that can be associated with a user's choice of a particular contraceptive option, J*_f,n_* (see File S4);
- legacy estrogenic load that can be associated with a user's choice of a particular contraceptive option, J*_L,n_* (see File S5);
- net estrogenic load averted by a population's collective use of contraception (see File S6);
- fraction of the net estrogenic load released over the course of all pregnancy events that results from those that are unintended, hence the failure and non-use of contraception among the user population (see File S7); and
- changes in associated estrogenic loading when users of EE_2_-OC users switch to other methods (see File S8).

Detailed summaries of data used, model parameters, and their corresponding literature sources are provided in File S9.

This work draws upon a wide variety of data sources in the environmental, family planning, reproductive health and clinical literature. Due to the very different natures of the data sources used, the parameterization of variables was accomplished using a multipronged approach. For variables whose parameterization drew upon environmental and clinical data, it was possible to capture uncertainty in parameterization by assigning appropriate distributions to the variables (see File S9). However, this was not possible for variables whose parameterization drew upon family planning literature. For such cases, parameterization of relevant variables was accomplished by either choosing the best possible parameterization for the variable or, where doubts existed as to what the best possible parameterization for a given variable was, conservative estimates of parameters were assigned. Overall, all estimates furnished through the modelling in this work were calculated using conservative values of inputs and parameters in order to arrive at readily defensible conclusions.

## Results and Discussion

### 1. Use of Contraceptives and their Associated Estrogen Loadings

In this section, an estimate is first furnished for the estrogenic load averted through the collective use of contraception (Section 1.1). An analysis is then conducted to evaluate and compare the estrogen loads that are attributable to the choice of individual contraceptive options (Section 1.2). Finally, given that some unintended pregnancies and, hence, births occur even with the use of currently available contraceptive choices, an evaluation is conducted to estimate the estrogenic load as it relates to pregnancy intent and to the effectiveness with which the population's current mix of contraceptive options is used (Section 1.3). All analyses presented are based on data reflecting trends in family planning in the USA.

#### 1.1 Net Estrogenic Load Averted by the Collective Use of Contraception

The collective use of contraception by a population averts a number of unintended pregnancies [43] and, hence at the very least, a load of natural steroidal estrogens to the environment. The approach used to estimate the total associated estrogenic mass averted due to the collective use of various contraception options by the population over a given year, *E_a_*, is detailed in File S6.

It is conservatively estimated that the collective use of contraception over the course of a given year in the USA averts 8.8 million unintended pregnancies (Eq. (S5)); i.e., 2.1, 5.0, 1.6 and 0.07 million unwanted births, abortions, fetal losses and ectopic pregnancies are averted, respectively, as estimated using Eq. (S5a) to (S5d) found in File S6. The total estrogenic load averted by the collective use of contraception in the USA over a given year (*E_a_*) can be estimated by adding the estrogenic load that would have been released over the course of 8.8 million unintended pregnancies (*E_p_*) to the estrogenic load arising from the legacies of 2.1 million *unwanted* births (*E_L_*) and subtracting from this sum the load that is directly released via excretions of users who currently use estrogen-based methods of contraception (*E_e_*) (see File S6).

Specifically, using Eq. (S4), the net quantity averted each year by the collective use of contraception in the USA, E_a_, is estimated to be 4.80 tonnes of E_2-eq_. This averted net load results from:

[0.66 tonnes of E_2-eq_ that would have been released over the course of 8.8 million averted pregnancies (E_p_)]
+[4.44 tonnes of E_2-eq_ estrogenic legacy of 2.1 million *unwanted* children whose births are averted (E_L_)]
[0.31 tonnes of E_2-eq_ estrogenic load directly released due to the current contraceptive mix (E_e_)]

The above estimates for *E_a_* and *E_p_* suggest that, on a per-pregnancy basis, the estrogenic load represented by an *unwanted* birth's legacy is substantially higher than that released over the course of an unintended pregnancy. The absolute magnitude with which the estrogenic load of an *unwanted* birth's legacy exceeds that released over the course of an unintended pregnancy is likely higher than what the above estimates would suggest since the manner with which we estimate the estrogenic legacy of an *unwanted* birth's legacy is highly conservative (see File S5). Moreover, it should be noted that the overall estimate of 4.80 tonnes of estrogen load averted is also highly conservative due to the chosen parameterization for a number of variables of the estimating equation (see File S6).

Overall, these results establish that the collectively use of contraception by the population of the USA averts a substantial estrogenic load from being released to the environment.

#### 1.2 Estrogenic Loads Associated with Individual Contraceptive Options

Based on the methodologies presented in Files S3 to S5 and using parameters evaluated from data collected in the USA (see File S9), the estrogenic load associated with the first-year use of each contraceptive option was estimated through the application of Eq. (1). The results are summarized in Table 1.

**Table 1:** Evaluation of Contraceptive Options: Annual probability (A_n_, %) with which unintended pregnancies are experienced by typical first-year users; estimated estrogenic loads associated with first year of use (*J_n_*,); proportion of resultant unintended pregnancies arising from inconsistent use; annualized cost of use; and rate of continuation of use of the option at the end of the first year.

Contraceptive Option (n)	A_n_ (a)	J_n_ (b) [J_d,n_ (c), J_f,n_ (d), J_L,n_ (e)]	Proportion of Unintended Pregnancies Due to Inconsistent Use(f)	Annualized Cost of Use(g) ^,^ (h)	Rate of Continuation of Use(a)
	%	mg of E_2-eq_/user•first year of use	%	$/user•yr	% of women after first year of use
No method used	85	304 [0, 43, 261]	Not applicable	948	
Spermicide	28	101 [0, 14, 87]	36	529	42
Fertility awareness-based methods	24	86 [0, 12, 74]	79	378	47
Withdrawal	22	79 [0, 11, 68]	82	403	46
Sponge (Parous women)	24	86 [0, 12, 74]	17	560	36
Sponge (Nulliparous women)	12	43 [0, 6, 37]	25	560	36
Female condom	21	75 [0, 11, 64]	76	535	41
Male condom	18	65 [0, 9, 56]	89	315	43
Diaphragm	12	43 [0, 6, 37]	50	434	57
EE_2_-based oral contraceptive	9	62 [29.5, 5, 28]	97	676	67
Progestin-only pill	9	33 [0, 5, 28]	97	n.a.(i)	67
Progesterone Injection	6	22 [0, 3, 19]	97	536	56
Copper IUD (ParaGard)	0.8	2.8 [0.0, 0.4, 2.4]	25	180	78
Levengesterol IUS (Mirena)	0.2	0.4 [0.0, 0.1, 0.3]	0	230	80
Female sterilization	0.5	1.2 [0.0, 0.2, 1.0]	0	596	100
Male sterilization	0.15	0.5 [0.0, 0.1, 0.5]	33	143	100
Implant	0.05	0.2 [0.00, 0.03, 0.15]	0	319	84

*J_n_* and all of its subcomponents are estimated on a pre-treatment basis.

(a) Trussell et al. [29];

(b) Estimated using Eq. 1;

(c) Estimated using Eq. (S1);

(d) Estimated using Eq. (S2);

(e) Estimated using Eq. (S3);

(f) Estimated using the using the method of Trussell et al. [64] as follows: (failure rate with typical use – failure rate with prefect use)/(failure rate with typical use) ×100, with failure rates as those reported by Trussell et al.[29];

(g) Annualized cost associated with the use of the contraceptive method over a time of horizon of 5 yrs., includes method related costs, cost of failures and the cost of side effects;

(h) From Trussell et al. [42],[47];

(i) Not available.

Of the commonly used options evaluated, the use of EE_2_-based oral contraceptives is the only one that results in a direct estrogen load (J*_d,n_*) since it involves the direct consumption and subsequent excretion of estrogenic content by the user. Since all contraceptive options fail, indirect loads J*_f,n_* (loads arising over the course of the unintended pregnancies) and J*_L,n_* (the estrogen legacy of the “extra” children that are the outcome of resultant unintended pregnancies) can be associated with the choice to use each option. As is evident from the results in Table 1, the choice to use certain non-estrogen based contraceptive options have quite significant indirect loads associated with their use. Such results indicate that relationship between the choice to use a given contraceptive option and its associated estrogenic load is a much more complex choice than broadly assumed in literature, whereby the choice to use estrogen-based options such as EE_2_-OC is the only one considered to impose estrogenic loading on the environment [26], [27]. These results demonstrate that certain non-estrogen based contraceptive choices have higher overall estrogenic loads associated with their use than the one simply associated with the choice to use EE_2_-OC.

When each contraceptive option's estimate for J*_n_* is compared to that of not using any method at all (i.e., J*_n_* for “no method” in the Table 1), the use of each and every method of contraception averts an associated estrogenic load from being released. This is consistent with the conclusion drawn earlier that the collective use of contraception, as opposed to using nothing at all, averts a substantial estrogenic load from being released. Hence, collectively and individually, the use of contraception prevents an estrogenic load from being released to the environment and, when evaluated in these terms, could be construed as being beneficial to the environment from the perspective of estrogen load when compared to no contraception at all. Since each averted unintended pregnancy also averts the added risk of maternal and neo-natal mortality [45], [46], the use of contraception, collectively and individually, in the USA can also be taken to avert a number of maternal and neo-natal mortalities. Overall, the use of various forms of contraception by the population of the USA, as opposed to using nothing at all, averts a substantial estrogenic load from being released, prevents a significant number of undesired family planning outcomes, and further averts a number of maternal and neo-natal deaths. Furthermore, Trussell [43] estimated that the collective use of contraception in the USA annually averts direct medical costs of nineteen billion USA dollars.

In order to perform a broadly applicable analysis of individual contraceptive choices, Table 1 also lists a number of additional parameters which are of importance from a family planning perspective. *A*_n_ can be viewed to be indicative of the efficacy with which each option *n* is typically used in the USA. In addition, the proportion of unintended pregnancies that are a result of inconsistent use of each option is also estimated. Note that this can be taken to be indicative of the fraction of the associated loads J*_f,n_* and J*_L,n_* for a given option *n* that could potentially be averted by ensuring consistent use of that option. Two additional data entries listed for each option are the cost associated with the use of each and the continuation rate expected one year after initiating the use of each specific option. The cost estimates [42], [47] include method-related costs, cost of failures, and the cost of associated side effects.

Among all contraceptive options listed in Table 1, including all those that are reversible and irreversible, the implant has the lowest estrogenic load associated with its use, since its failure rate (reflected by *A_n_*) is the lowest of all contraceptive options. Also, of all reversible contraceptive options, the implant, probably due to its inherent nature, also has one of the highest continuation rates (see Table 1). However, this contraceptive option has been adopted by a relatively minor fraction of the potential user population in the USA [48], [49].

EE_2_-OC and the male condoms are the two most common forms of reversible contraception used in the USA [49]. Of the two, EE_2_-OC is more effective in preventing unintended pregnancies, results in a lower associated estrogenic load, and is more likely to be continued to be used once adopted. Particularly striking is the fact that inconsistent use of EE_2_-OC and the male condom results in 97% and 89% of all unintended pregnancies experienced by first-year users of the two options, respectively. Hence, the associated estrogenic loads J*_f,n_* and J*_L,n_* for these two options can be substantial reduced by improving the consistency with which they are typically used in the USA.

The copper IUD is the most cost effective of all reversible and irreversible contraceptive options [42], [47]. Furthermore, of all reversible contraceptive options, it results in one of the lowest associated estrogenic loads (see Table 1). Moreover, it is highly effective in preventing unintended pregnancies among its users and has one of the highest continuation rates of all reversible contraceptive options. Hence, the copper IUD performs exceptionally well on the various criteria against which the use of contraceptive options are evaluated in Table 1. This contraceptive option along with the use of Levonorgestrel-based intrauterine system (IUS) has, in recent years, experienced a renewed interest in use amongst contraceptive users in the USA [48]. Consider that, amongst users of contraception in the USA, the use of these two options has increased from 2.0% in 2002 to 8.8% in 2009 [48].

#### 1.3 Estrogenic Loads Resulting from Unintended Pregnancies

**Figure 3:**
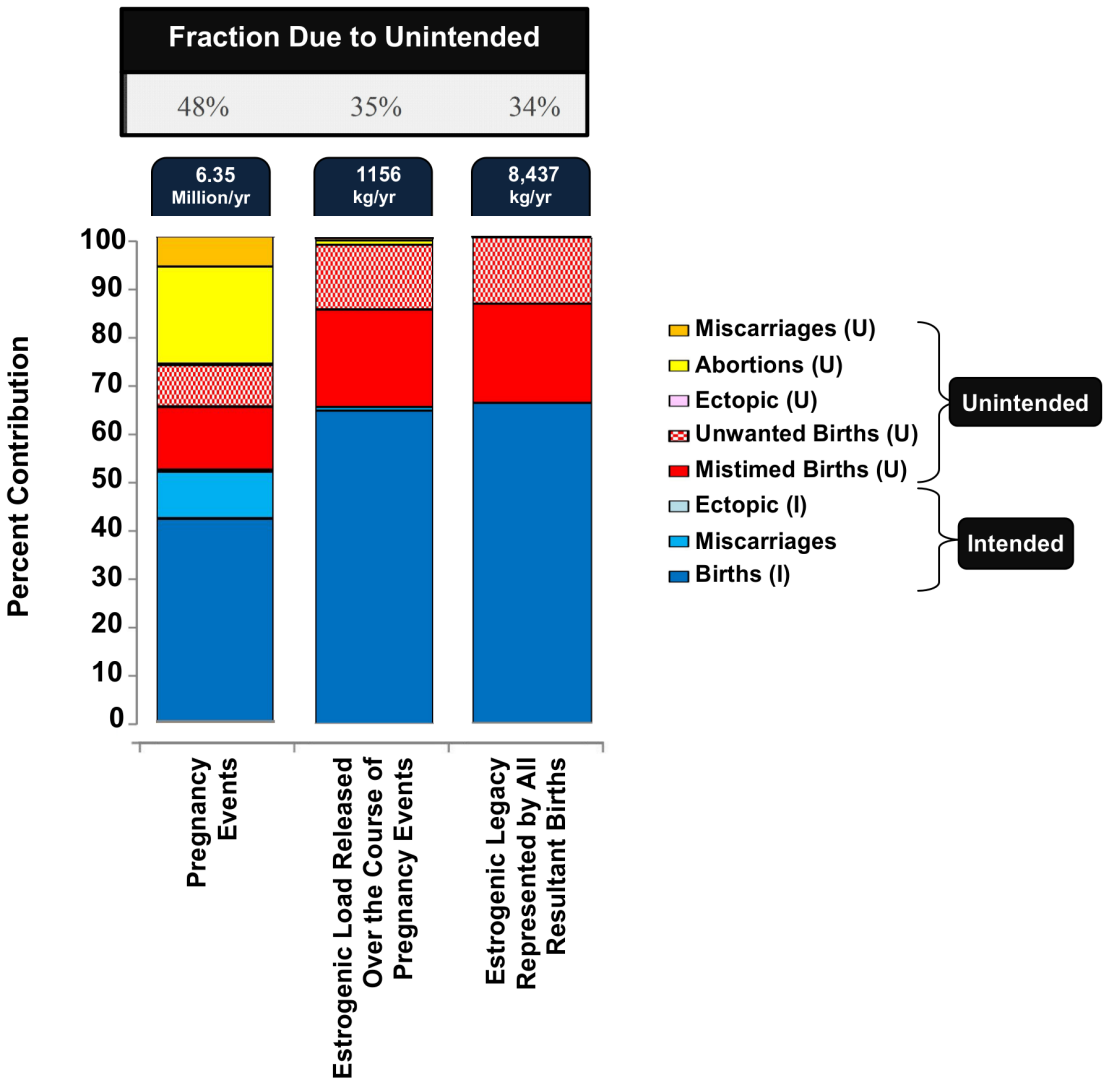
Relative contributions of intended (I) and unintended (U) pregnancy events to the total number of pregnancies, the estrogenic load released over the course of all pregnancies, and the estrogenic legacy represented by all resultant births. Refer to S7 to see how the various contributions were estimated.

Estimates suggest that nearly half of the 6.35 million pregnancy events experienced annually in the USA are unintended (see Figure 3) [30], [31]. Hence, it is particularly interesting to evaluate how the estrogenic load that is released due to, and over the course, of these events relates to pregnancy intent and, hence, to the effectiveness with which various contraceptive methods are currently used in the USA.

The various contributions to estrogenic loads of each of the major pregnancy events were estimated in terms of estradiol equivalents (E_2-eq_). The results summarized in Figure 3 suggests that, over the course of these 6.35 million pregnancy events, an estimated 1.2 tonnes of E_2-eq_ is released; however, only 35% of this load (i.e., 0.40 tonnes) arises from unintended pregnancies and, hence, from the failure and non-use of contraception. There are two reasons why an average unintended pregnancy event, when compared to an intended one, leads to the release of less estrogenic mass. First, only 45% of unintended pregnancies end up in the outcome of birth, in contrast to 81% of intended pregnancies [30]. Additionally, a pregnancy resulting in the outcome of birth leads to the release of an E_2-eq_ load that is on average 20, 25 and 32 times higher than the levels released had the pregnancy instead concluded in the outcome of spontaneous abortion, induced abortion and ectopic pregnancy, respectively (see File S1).

Of the 6.35 million pregnancy events experienced in the USA each year, 4.02 million events culminate in the birth of a child [30], [31]. Hence, each year, 4.02 million legacies are born to the population of the USA and these can be estimated to at least represent an estrogenic legacy load of 8.4 tonnes of E_2-eq_ (see Figure 3). However, as indicated earlier, since an unintended pregnancy, when compared to an intended one, is less likely to end up in the outcome of birth, only 34% of the estrogenic legacy load results from unintended pregnancies (Figure 3). Hence, the failure and the non-use of contraception by the population of the USA results in 34% of the estrogenic legacy birthed each year.

### 2. Implications of Discontinuing the Use of EE_2_-based Oral Contraceptives

To-date there has been a tendency in the literature to only account for the direct estrogen load associated with a given contraceptive method (J*_d,n_*). Due to this and also the growing evidence of the impact of ethinylestradiol, EE_2_, on aquatic species [12], [33], [50], [66], particular focus has been placed on EE_2_-based contraceptives [32]. This has led some to suggest that, because of its environmental impacts and/or the high costs associated with the treatment of wastes containing this estrogen, the use of EE_2_-based contraceptives should be a subject of further discussion [27], [32]. Such analyses and, more broadly, almost all equivalent literature concerning estrogen loads on the environment, fail to recognize the full extent of the relationships between a user's choice to use a given contraceptive option and estrogen loading to the environment. A more informative analysis would be one that aims to understand the trade-offs involved in the choice to use EE_2_-OC. To this end, we will explore how the estrogenic flows would be expected to change should a group of EE_2_-OC users discontinue the use of their method by switching to a range of other contraceptive methods that are currently available to them or by abandoning the use of contraception altogether. This assessment is performed by conducting a scenario analysis. Specifically, in Section 2.1, the estrogenic load under the *Status quo* scenario for a unit population of a 1,000 first-year EE_2_-OC users is compared to a *Discontinue EE_2_* scenario, the aim of which is to model the estrogenic load associated with the most likely contraceptive choices made by the user group upon discontinuing the use of the EE_2_-OC; i.e., upon discontinuing the use of EE_2_-OC the switching population is expected to either adopt other available contraceptive options or discontinue the use of contraception altogether. The contraceptive choices made by the switching population are modeled using the data of Rosenberg and Waugh [51] who reported the contraceptive mix adopted by those in the USA who for various reasons discontinued the use of EE_2_-OC but still wanted to prevent a pregnancy.

In addition to the *Discontinue EE_2_-OC* scenario, we also consider in Section 2.2 three other explorative scenarios in which the population of a 1000 EE_2_-OC users, *P_s_*, is modeled to switch, as per the suggestion of Wise et al. [26], to using male condoms, diaphragms or copper IUDs. While these are not considered to be likely scenarios, they provide a basis for comparing the estrogenic loads arising from particular contraceptive choices. Among these, the scenarios that explore the switch to the use of male condoms and to the use of copper IUDs are particularly interesting. The male condom, after the use of EE_2_-OC, is the most common form of reversible contraception used by couples in the USA [49]. The use of copper IUDs is not only the most cost-effective reversible contraceptive method [42] but is also one of the most effective in preventing pregnancies [29].

Recently, an oral contraceptive preparation that uses estradiol, E_2_, as the active ingredient has been approved for sale in the USA [15], [21]. It is reasonable to assume that some users of EE_2_-OC would switch to this new preparation. Hence, it is of particular interest to evaluate how the estrogenic flows to the environment would change when a group of EE_2_-OC users adopts this unique estradiol-based oral contraceptive (E_2_-OC). The results of this analysis are detailed in Section 2.3.

#### 2.1 Scenario

**Figure 4:**
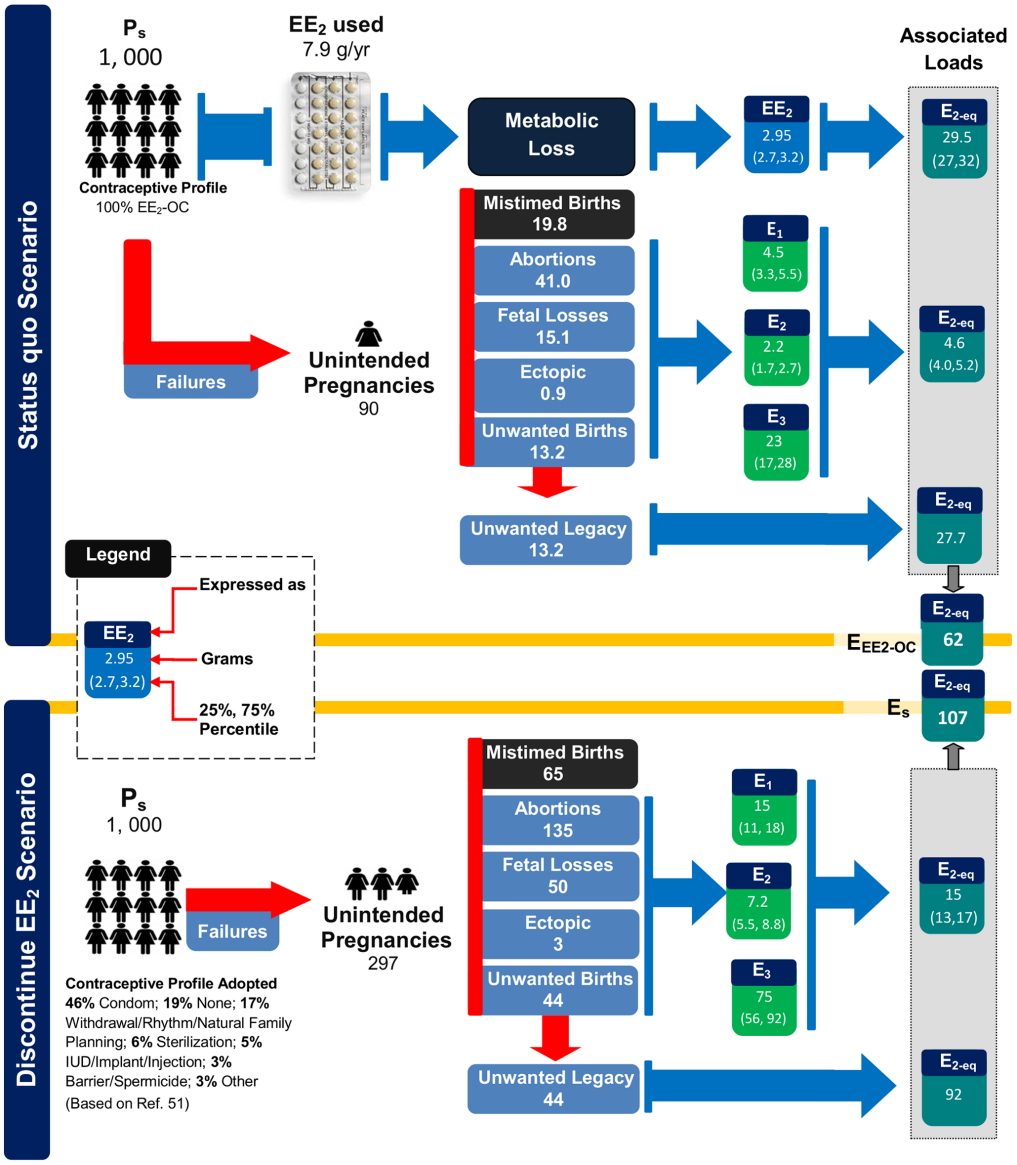
Changes in associated loads of steroidal estrogens when a unit of population of 1,000 first users of EE_2_ based oral contraceptive switch to other contraceptive options. The total estrogen load associated with oral contraceptive use (EE_2_-OC) was estimated using Eq. (S6). The total estrogen load associated with those who discontinue the use of oral contraception (E_s_) was estimated using Eq. (S7).

Changes in Estrogenic Load upon Discontinuation of EE2-OC. It was established above that the use of EE_2_-OC averts an estrogenic load from being released to the environment when compared to using no contraceptive method at all. However, a particularly valuable and, perhaps, more realistic evaluation is one that compares the changes in loads of associated estrogens when EE_2_-OC users discontinue the use of their method by switching to other contraceptive methods or by abandoning the use of contraception altogether. Such an assessment is performed here by conducting a scenario analysis as described in detail in File S8. The results of the analysis are summarized in Figure 4.

Overall, it is estimated that, when first-year users of EE_2_-OC discontinue the use of their option, they will experience unintended pregnancies at a rate of 297 per 1000 users per year, a level that is nearly 3.3 times higher than the levels they would have experienced had they continued using EE_2_-OC (see Figure 4). Note that the estimated unintended pregnancy rate for those that discontinue the use of EE_2_-OC is particularly sensitive to the fraction of those that abandon the use of contraception altogether. That is, it is estimated that a group of approximately 190 persons who completely abandon the use of EE_2_-OC in favor of no contraception at all will contribute to 162 (or 54%) of the anticipated 297 unintended pregnancies resulting from the original 1000 EE_2_-OC users who switched to other options.

The scenario analysis suggests that under the *Status quo* scenario shown in Figure 4, 62 grams of E_2-eq_/yr (*N_EE2-OC_*) can be associated with the representative user population of 1,000 EE_2_-OC users. Of this total load, 48% results from the direct excretions of the user population, another 7% is released over the course of unintended pregnancies experienced by the user population, and the remaining 45% is the estrogenic load represented by the legacies of unwanted children born to the representative user population. Upon discontinuing the use of EE_2_-OC, it is estimated that the user population's total estrogenic load (*N_s_*) will increase 1.7-fold to 107 grams of E_2-eq_/yr, of which 14% would be released over the course of 297 unintended pregnancies experienced by the discontinuing population and the remaining 86% would be the estrogenic load represented by the legacies of unwanted children expected to be born to that population. If the various contributions in Eq. (1) to the loads for the *Status quo* and *Discontinue EE_2_* scenarios are compared, it can be seen that the increase in total estrogenic load upon discontinuing the use of EE_2_-OC is driven by a substantial increase in the legacy contribution (*J_L,n_*). Further, over and above the 1.7-fold increase in estrogenic load experienced upon discontinuing EE_2_-OC use, the user population also experiences undesired family outcomes such as unintended pregnancies, abortions, miscarriages and unplanned births at levels that are approximately 3.3 times higher. Similarly, because the contraceptive needs of the parents could not be met, the number of extra births also increases by 3.3 times and the number of ectopic pregnancies experienced by the user population also increases by an estimated 3.4 times. Further, recognizing that each additional unintended pregnancy presents an added risk of maternal and neo-natal mortality [45], [46], [52], upon discontinuing the use of EE_2_-OC, the user population would be expected to experience far higher rates of maternal and neo-natal mortality. Hence, whether the assessment of contraceptive options is performed by considering estrogenic loading, the number of undesired family planning events experienced, or maternal and neo-natal mortality rates, the continued use of EE_2_-OC appears to be a much-preferred option over discontinuing its use and switching to a range of other options. Notably, nearly one-third of the EE_2_-OC users discontinue the use of their option within the first year [29].

#### 2.2 Scenario

EE2-OC Users Switch to Specific Non-estrogen-based Contraceptive Options. The above assertion that discontinuing the use of an EE_2_-based oral contraceptive will result in an increase in total estrogen load into the environment hinges on the assumption that users will switch to a particular set of alternative options, as described by the data of Rosenberg and Waugh [51]. However, this analysis does not directly reveal the relative merits of individual contraceptive options that can be used in place of EE_2_-based oral contraceptives. For this reason, and to concurrently evaluate the suggestion of Wise et al. [26], consider three explorative scenarios where, perhaps unrealistically, the unit population of a 1,000 EE_2_-OC users are modeled as having discontinued the use of EE_2_-OC by entirely switching to the use of either the male condom, the diaphragm, or the copper IUD. The results for each of these three scenarios are summarized in figures in Files S10, S11 and S12, respectively.

If the entire user population switches to the use of male condoms, the associated estrogenic load would be slightly higher (specifically 1.05 times); moreover the level of unintended pregnancies, abortions, miscarriages and unplanned births experienced by the user population would approximately double (see File S10). While on one hand, the switch to diaphragms results in some modest gains with respect to reduced estrogenic loading, it is also expected to lead to an unacceptable increase in the rate at which the user population experiences undesired family planning events (see File S11). In contrast, should the entire user population switch to the use of copper IUDs, not only would their associated estrogenic load be approximately one-twentieth compared to the *Status quo* scenario, the user population would experience significantly fewer adverse personal, social and public health outcomes (see File S12). Consider that, upon switching to the copper IUD, the user population would be expected to experience unintended pregnancies, abortions, miscarriages and unplanned births at rates that are one-twelfth the levels they would have experienced had they continued using EE_2_-OC (see File S12). In addition to these already substantial benefits, for each user that switches from the use of EE_2_-OC to a copper IUD, the costs associated with the chosen method of contraception would be an estimated US $500 less for each year of use [42], [47]. This gain in cost effectiveness is the highest that can be achieved for an EE_2_-OC user who wishes to switch to another reversible contraceptive option, since the copper IUD is the most cost-effective reversible contraceptive option currently available to the population of the USA [42], [47].

The results of the above scenario analyses indicate that whether the switch of an EE_2_-OC user population to alternative modes of contraception leads to an increase or decrease in estrogenic load on the environment is inherently dependent on the alternate forms of contraception chosen by the user population. Note that, even though the estrogenic load of the user population is expected to increase significantly for those who currently discontinue the use of EE_2_-OC as reported above (see Section 2.1), this outcome is inherently dependent on the mix of contraceptive choices chosen by users upon discontinuation. Hence, this would have to be reevaluated should these patterns change in the future. In addition, the results arising from the scenario analyses for users who switch to the male condom and diaphragms clearly suggest that the choice to use a given contraceptive option, or any recommendations that may influence that choice (e.g., the suggestions of Wise et al. [26]), should not only be evaluated on the basis of their associated estrogenic loads but also by considering broader public health and family planning implications for the user population.

#### 2.3 Scenario

Users of EE2-OC Switch to E2-OC. Recently, an E_2_-based oral contraceptive has been made available in the USA and, hence, it would be of interest to evaluate how the estrogen loading changes when a user switches to this form of contraception from the prior use of EE_2_-OC.

The analysis presented in File S13 suggests that the associated estrogenic load of an E_2_-OC user is nearly 2.2 times higher that of an EE_2_-OC user. This increase is a direct result of 3.6–fold increase in the direct estrogenic load, J*_d,n_*, since the contributions J*_f,n_* and J*_d,n_* for each of the two options, based on current knowledge, would be expected to be similar if not identical (See File S8).

### 3. Impact of Contraceptive Choices on the Net Steroidal Estrogenic Loads

The results obtained above clearly suggest that the use of certain contraceptive options have a lower total estrogenic load associated with their use than others. Given this, a very important and pragmatic question must be raised; that is, since certain contraceptive options have lower estrogenic loads associated with their use than others, what absolute impact can contraceptive users in the USA have on the net estrogenic load released to the environment by switching to contraceptive choices that result in lower loads of estrogens?

To answer this question, consider the estimates presented in Figure 3. Overall, the failure of contraceptive options and also the non-use of contraception by the population of the USA over a given year represent an estimated total estrogenic load of 3.3 tonnes of E_2-eq_, of which 0.40 tonnes is released over the course of 3.05 million unintended pregnancies and another 2.9 tonnes is the estrogenic load represented by the legacies of 1.37 million unintended births (see Figure 3). Note, however, that this entire load would not be eliminated in the event that the family planning needs of the population of the USA can be fully met. Specifically, a substantial fraction of these loads arises from those pregnancies that will result in the outcome of *mistimed* births. As argued earlier, such pregnancies represent a time-displaced estrogenic load and, hence, the estrogen release cannot be mitigated by meeting the family planning needs of the experiencing population. Specifically, 0.23 tonnes of the 0.40 tonne load and 1.7 tonnes of the 2.9 tonne load result from those unintended pregnancies that end in an outcome of *mistimed* birth. Hence, by fully meeting the contraceptive needs of the population of the USA, an estrogenic load of 1.3 tonnes of E_2-eq_ (i.e., (2.9–1.7) tonnes +(0.40−0.23) tonnes) can potentially be averted. Viewed another way, the failure and the non-use of contraception by the population of the USA currently represents a potentially preventable annual estrogenic load of 1.3 tonnes of E_2-eq_. Further, if it is assumed that the contraceptive needs of contraceptive users in the USA can be fully met without the use of EE_2_-OC, the release of an additional 0.31 tonnes of E_2-eq_ (see File S1) can be averted.

The potentially preventable estrogenic load of 1.3 tonnes of E_2-eq_ should be compared to the net steroidal estrogenic load in the USA for a given year to answer the question raised above. The net steroidal estrogenic load in the USA in a given year is estimated to be 10.4 tonnes of E_2-eq_, of which 2.0 tonnes of E_2-eq_ are directly released via excretions of users (see File S1) and the remainder of 8.4 tonnes of E_2-eq_ is the estrogenic legacy that is born to them that year (see Figure 3). Therefore, by fully meeting the contraceptive needs of the population of the USA through a contraceptive option that is not estrogen-based, at most 13% (i.e., (1.3 tonnes +0.3 tonnes)/10.4 tonnes ×100%) of the estrogenic load in the USA can be averted in a given year. This fraction is the absolute maximum that can be prevented since it is inherently assumed that all users of contraception switch to methods that fully meet their needs. Note that this is an idealized condition since such methods do not exist given that even the most effective methods (e.g., implant, copper IUD and IUS) also fail, albeit at very low rates [29]. Further, consider that in the estimate made here for the annual net steroidal estrogenic load in the USA, the fraction that is contributed by the release of equine estrogens has not been considered. This is due to considerable data gaps that exist for the release and the environmental relevance of such estrogens (see File S14). The preliminary evaluation presented in File S14 suggests that the release of such estrogens could be a minor but significant contributor to net steroidal estrogenic load in the USA each year.

Overall, the maximal estimate of 13% of the annual estrogenic load in the USA that can be averted through alternative forms of contraception suggests that the issue of estrogenic loading to the environment cannot be solved solely by meeting the population's contraceptive needs. That being said, significant gains in terms of reduced environmental impacts could be achieved by improving the level with which the contraceptive needs of the population are met.

Since the potentially preventable load in the USA of 1.3 tonnes of E_2-eq_ estimated above results from users that are either experiencing failure or not using contraception altogether, it is of further interest to establish the relative impact of each user type on the estimated load. Before this is done, it is important to note that there are three types of users of contraception who experience unintended pregnancies: consistent users, inconsistent users, and non-users of contraception. Data from the Guttmacher Institute [53] can be used to estimate that non-users and inconsistent users are 42 and 29 times more likely, respectively, to experience an unintended pregnancy than consistent users. Further, consider that 52% and 43% of all unintended pregnancies experienced in the USA are by non-users and inconsistent users of contraception, respectively [53]. Hence, the estrogenic load of 1.3 tonnes of E_2-eq_, estimated above largely results from the non-use and inconsistent use of contraception. Thus, gains can be made with respect to the estrogenic loading of the population in the USA by improving the consistency of use among inconsistent users and by improving the adoption of contraception among those who are currently at a risk of experiencing pregnancies but do not use any form of contraception. The former can be achieved by either directly improving the typical efficacy with which users use their chosen contraceptive options and/or, more plausibly, by encouraging users of those options that have high typical failure rates (e.g., withdrawal) to switch to those methods that have significantly lower typical use failure rates (e.g., copper IUD, IUS or the implant).

## Study Limitations

The results of this study are intended to inform discussions concerning the relationships between contraception options and their estrogenic impacts on the environment. However, in the interest of clarity, it is also important to point out some limitation to this study, as follows.

With respect to the conclusion drawn in Section 2.1, it is important to note that even though the associated estrogenic load of users is expected to be higher upon discontinuing EE_2_-OC use, the higher load of estrogens in the environment for the *Discontinue EE_2_* scenario is expected to be considerably less persistent than that released under the *Status Quo* scenario. This assertion results from the recognition that EE_2_ has been reported to be substantially more persistent than natural estrogens [25]. That being said, it is worth noting that emerging data suggests that a previously unrecognized photolysis product of estrone (i.e., lumiestrone) may not only be estrogenic but also persistent [54]–[56]. However, the environmental occurrence and relevance of this photolysis product is not yet fully understood. Hence, even though well-established data [17], [24], [25] suggests that the lesser estrogen load of the *Status quo* scenario is likely to be more persistent, this assertion would need to be revaluated once sufficient data on the environmental fate, occurrence and relevance of lumiestrone becomes available.

Similarly, with respect to the conclusion drawn in Section 2.3 that the associated loading of a previous EE_2_-OC user increases 2.2-fold when she switches to the use of E_2_-OC, one must also consider that, although lower in quantity, the total associated estrogenic load of an EE_2_-OC user may be more persistent than the one released by the user of E_2_-OC. That is, the excretion of EE_2_ by the users of EE_2_-OC are replaced by releases of E_1_, E_2_ and E_3_ by the users of E_2_-OC, all of which are less persistent than EE_2_ [17], [24], [25]. Therefore, before a definitive conclusion concerning the relative impacts of these estrogens can be made, a better understanding of the fate and relative environmental relevance of lumiestrone is required.

Although the focus of this research is to develop a better understanding of the relationship between contraceptive choices and the associated estrogenic loads, it is important to note that the choice to use a given a contraceptive option also has implication for the release of gestagens to the environment. Clearly, a user's choice to use a progestin-based contraceptive option (e.g., EE_2_-OC, E_2_-OC, progestin-only pill, Depo-Provera, levonorgestrel-IUS, and the implant) results in the direct release of a number of gestagens to the environment that is analogous to J*_d,n_* for estrogens. Moreover, similar to the situation when evaluating the total associated estrogenic load, the relationships are more complex than the simple consideration that the use of only gestagen-based options only leads to their direct release to the environment. In fact, analogous to the contributions J*_f,n_* and J*_L,n_* discussed above for estrogens, the choice to use every single contraceptive option has at least two indirect loads of gestagens associated with their use. First, the gestagen load analogous to J*_f,n_* results from the recognition that every contraceptive option has a failure rate associated with its use and the resultant unintended pregnancy leads to an increased release of progesterone [57], a natural gestagen, to the environment. And, second, the gestagen load analogous to J*_L,n_* results from the recognition that, had a chosen contraceptive option not failed and further the resultant unintended pregnancy not resulted in the outcome of unwanted pregnancy, the unwanted child's legacy load of gestagens would not have resulted. Therefore, overall, every contraceptive option comes with an associated gestagenic load. Unfortunately, since the eco-toxicological potential for only a handful of gestagens has been explored in sufficient detail in literature [58]–[63], it is presently not possible to quantify on an equivalents basis the gestagenic load associated with the use of any given contraceptive option. Due to the absence of such data, it is also presently not possible to suggest whether the collective use of contraception or the use of a given contraceptive options averts a gestagenic load from being released to the environment.

Similarly, for the analysis presented in Section 2.3 the use of E_2_-OC leads to the direct release of dienogest, a progestin that is only used in the USA with E_2_-OC, while the users of EE_2_-OC leads to the direct release of a range of other progestins [15]. However, again due to lack of sufficient eco-toxicological data for all progestins directly released by users of the two forms of oral contraceptive, it presently not possible to determine whether the choice of using E_2_-OC over EE_2_-OC will result in a higher or a lower associated gestagenic load being released to the environment.

## Conclusions

This research has focussed on developing an understanding of the relationships between the choice to use a given contraceptive option and the associated loads of steroidal estrogens on the environment. The conceptual approaches and models developed in this study were applied to the population of contraception users in the USA to establish the following:

- The use of each contraceptive option, even when it is not estrogen-based, has a load of steroidal estrogens associated with its use. However, when compared to the estrogenic load associated with the use of “no contraception” at all, the use of every contraceptive option, including those that are estrogen-based, prevents an estrogenic load from being released to the environment.
- The collective use of contraception in the USA over a given year conservatively averts 8.8 million unintended pregnancies and, in doing so, averts the release to the environment of an estimated estrogenic mass of 4.8 tonnes, expressed in estradiol equivalents.
- Almost half of all pregnancy events experienced in the USA in a given year are a result of contraception failure and the non-use of contraception. However, only 35% of the estrogenic load released over the course of resultant pregnancies and 34% the estrogenic load represented by all resultant legacies are a result of contraception failure and the non-use of contraception.
- When current users of EE_2_-based oral contraceptives discontinue the use of their option, not only is it expected that their overall estrogenic load increases 1.7-fold but they also would be expected to experience undesired family planning outcomes at a rate that is 3.3 times greater.
- Additional analyses were conducted on three idealized scenarios in which a group of EE_2_-based oral contraceptive users were modelled as having switched entirely to the use of male condoms or diaphragms or copper IUDs. The results arising from all of these scenarios suggests that the outcome of whether higher or lower estrogen loads can be associated with the discontinuation of the use of EE_2_-based oral contraceptives ultimately depends on the typical failure rates of the options adopted following discontinuation.
- In addition, explorative scenarios in which users were assumed to switch from EE_2_-based contraceptives to alternative contraceptive options indicated that the choice of whether to use one contraceptive option versus another should not be assessed based on estrogenic load considerations alone but also by considering broader public health and family planning implications. When evaluated with such a broad framework, the switch from the use of EE_2_-OC to that of copper IUDs seemed particularly interesting because its use is associated with an estrogenic load that is approximately one-twentieth of that associated with the use of EE_2_-OC. However, any such recommendation should also be sensitive to cultural, physical, emotional, and psychological implications for the user.
- Another scenario considered was the plausible switch of users of EE_2_-based oral contraceptives to the newly approved E_2_-based oral contraceptive. Upon switching to this alternative estrogen-based contraceptive, the associated estrogen load of the user would be expected to increase by approximately 2.2-fold.
- At most, 13% of the net annual estrogenic load to the environment can be averted by fully meeting the contraceptive needs of the population of the USA. Hence, the issue of estrogen loading cannot solely be addressed by meeting the contraceptive needs of the population.

## Supporting Information

File S1 **Estimated Anthropogenic Steroidal Estrogen Loads in the USA circa 2002.**
(DOC)

File S2 **Eco-toxicological Potency of Steroidal Estrogens.**
(DOC)

File S3 **Modeling the Steroidal Estrogen Load Released Directly Via the Use of a Particular Contraceptive Option (J_d,n_).**
(DOC)

File S4 **Modeling the Load of Natural Estrogens Released over the Course of Unintended Pregnancies that can be Associated with a User's Choice to Use a Particular Contraceptive Option (J_f,n_).**
(DOC)

File S5 **Modeling the Legacy Estrogenic Load Arising from an Unwanted Birth and can be Associated with a User's Choice to Use a Particular Contraceptive Option (J_L,n_).**
(DOC)

File S6 **Pregnancy Events and Estrogenic Load Averted by a Population's Collective Use of Contraception.**
(DOC)

File S7 **Estimating Relative Contributions as Shown in** **Figure 3** **(Main Body).**
(DOC)

File S8 **Modeling Estrogen Loads Arising from Changes in Contraceptive Use.**
(DOC)

File S9 **Nomenclature and Specific Estimates for All Variables Used.**
(DOC)

File S10 **Changes in Flows of Estrogens When Users of EE_2-OC_ Switch to Male Condoms.**
(DOC)

File S11 **Changes in Flows of Estrogens When Users of EE_2-OC_ Switch to Diaphragms.**
(DOC)

File S12 **Changes in Flows of Estrogens When Users of EE_2-OC_ Switch to Copper IUDs.**
(DOC)

File S13 **Flow of Estrogens Associated with the Use of E_2-OC_.**
(DOC)

File S14 **Excretion of Equine Estrogens and their Relevance.**
(DOC)

File S15 **References for All Supporting Information Sections.**
(DOC)
