# Supplementary material for: Contraceptive Options and Their Associated Estrogenic Environmental Loads: Relationships and Trade-Offs
Source: PLoS One. 2014 Mar 26;9(3):e92630. doi: 10.1371/journal.pone.0092630 (PMC3966801; doi:10.1371/journal.pone.0092630)
Supplement: File S1 — Estimated Anthropogenic Steroidal Estrogen Loads in the USA circa 2002. (DOC) [file pone.0092630.s001.doc]

# S1 Estimated Anthropogenic Steroidal Estrogen Loads in the USA circa 2002

**Table S1** Estimated Net Load of Steroidal Estrogens Via Domestic Wastewater Treatment Plants circa 2002.

| **Population cohorts/**  **Pregnancy outcomes** | **Number** |  | **Daily per capita excretion** | | | |  | **Excretion per pregnancy outcome** | | |  | **Net contribution** | | | | | **Pre-treatment Load** | **Post-treatment Load** | **40d Post Release Load^(s)^** |
| --- | --- | --- | --- | --- | --- | --- | --- | --- | --- | --- | --- | --- | --- | --- | --- | --- | --- | --- | --- |
|  |  |  | **E_1_** | **E_2_** | | **E_3_** |  | **E_1_** | **E_2_** | **E_3_** |  | **E_1_^(d)^** | | **E_2_^(d)^** | **E_3_^(d)^** | **EE_2_** | **E_2 –Eq_^(e)^** | **E_2 –Eq_^(e),(t)^** | **E_2 –Eq_^(e),(u)^** |
|  |  |  | **µg/cap·d** | | | |  | **mg/outcome** | | |  | **kg/yr** | | | | | **kg/yr** | | |
| ***Endogenous*** |  |  |  | |  |  |  |  |  |  |  |  |  | |  |  |  |  |  |
| **Females** |  |  |  | |  |  |  |  |  |  |  |  |  | |  |  |  |  |  |
| Prepubescent girls | 24,433,426 | **(a),(b)** | 0.08 | | 0.06 | 0.2 | **(c)** |  |  |  |  | 0.7 | 0.6 | | 1.9 |  | 0.9 | 0.1 | 0.0 |
| Menstruating women  not on OC | 64,701,425 | **(m)** | 8.7 | | 4.4 | 9.4 | **(r)** |  |  |  |  | 205 | 104 | | 223 |  | 182 | 25 | 0.0 |
| Menstruating women on OC | 11,635,029 | **(g)** | 3.1 | | 1.2 | 2.5 | **(r)** |  |  |  |  | 13 | 5 | | 11 |  | 10 | 1 | 0.0 |
| Menopausal women | 42,205,554 | **(h),(b)** | 1.7 | | 0.8 | 1.9 | **(r)** |  |  |  |  | 26 | 12 | | 30 |  | 22 | 3 | 0.0 |
| Pregnancy – Births | 4,022,000 | **(i)** |  | |  |  |  | 259 | 134 | 1525 | **(j)** | 1040**^(n)^** | 539**^(n)^** | | 6132**^(n)^** |  | 1131 | 135 | 0.0 |
| Pregnancy –  Induced abortions | 1,269,000 | **(i)** |  | |  |  |  | 12 | 4 | 22 | **(k)** | 15(**^n)^** | 5**^(n)^** | | 28**^(n)^** |  | 11 | 2 | 0.0 |
| Pregnancy –  Spontaneous abortions | 1,016,206 | **(l)** |  | |  |  |  | 18 | 6 | 45 | **(k)** | 19(**^n)^** | 6**^(n)^** | | 46(**^n)^** |  | 14 | 2 | 0.0 |
| Pregnancy – Ectopic | 40,621 | **(l)** |  | |  |  |  | 15 | 5 | 29 | **(k)** | 1(**^n)^** | 0.2(**^n)^** | | 1(**^n)^** |  | 0.4 | 0.1 | 0.0 |
|  |  |  |  | |  |  |  |  |  |  |  |  |  | |  |  |  |  | 0 |
| **Males** |  |  |  | |  |  |  |  |  |  |  |  |  | |  |  |  |  |  |
| Prepubescent boys | 24,924,262 | **(p),(b)** | 0.08 | | 0.06 | 0.2 | (c) |  |  |  |  | 0.7 | 0.5 | | 1.8 |  | 0.9 | 0.1 | 0.0 |
| Males | 116,306,297 | **(q),(b)** | 3.6 | | 2.13 | 2.4 | (r) |  |  |  |  | 153 | 90 | | 102 |  | 145 | 20 | 0.0 |
|  |  |  |  | |  |  |  |  |  |  |  |  |  | |  |  |  |  |  |
| ***Exogenous*** |  |  |  | |  |  |  |  |  |  |  |  |  | |  |  |  |  |  |
| Women on HRT**^(o)^** |  |  |  | |  |  |  |  |  |  |  | 232 | 67 | | 38 |  | 146 | 24 | 0.0 |
| Women on OC**^(f)^** |  |  |  | |  |  |  |  |  |  |  |  |  | |  | 30.7 | 307 | 49 | 3.0 |
|  |  |  |  | |  |  |  |  |  |  |  |  |  | |  | |  |  |  |
|  |  |  |  | |  |  |  |  |  |  |  |  |  | | E_2 –Eq_ (Total) | | **1,970** | **261** | **3.0** |

OC = Oral Contraceptive, HRT = Hormone Replacement Therapy

**Footnotes**

**(a)** Women less than the age of menarche of 12.5 years^1^.

**(b)** Estimated using the data reported by US Census data^2^ for the year 2002.

**(c)** Shi et al.^3^.

**(d)** Unless otherwise indicated, estimated as follows: daily per capita excretion (µg/cap·d) × cohort population × 365 (d/yr) × 1/10^9^ (kg/µg).

**(e)** E_2–Eq_ = [E_1_]/3 + [E_2_] + [E_3_]/25+10[EE_2_]; where: [E_2-Eq_], [E_1_], [E_2_], [E_3_] and [EE_2_] are the mass loads of Estradiol equivalents, estrone, estradiol, estriol and ethyinlestradiol, respectively. See Section S2 for justification for the potencies (i.e., 1/3, 1, 1/25 and 10) used within this equation.

**(f)** Estimated by multiplying the annual consumption of EE_2_ in the USA of 82.4 kg^4^ by fraction of the drug that is excreted as unchanged EE_2_ and its conjugates (estimated in Section S3).

**(g)** Mosher and Jones^5^.

**(h)** Women aged more than the mean menopausal age of 51 years^6^.

**(i)** Ventura et al.^7^

**(j)** The urinary contribution was estimated by firstly multiplying data reported by Berg and Kuss^8^, which reported the mean excretions curves of natural estrogens for women of various gestational ages in the units of grams of estrogen per gram of creatinine by the average daily excretion of creatinine (g of creatinine/24hr) by pregnant women^9^. The resultant excretion curves were integrated over the duration of a pregnancy that results in the outcome of birth^41^ to arrive at cumulative levels of the various natural estrogens that are eliminated via the urinary release of pregnant women who end up giving birth. Thus far, only a handful of analyses^11,12^ have been performed for the fecal release of natural estrogens by pregnant women. Therefore, as the best approximation, the limited fecal data^11,12^ that was available was used to estimate fecal-to-urinary excretion ratios for each natural estrogen. Subsequently, the estimated ratios for each natural estrogen were multiplied by their respective the urinary levels to arrive at best possible estimates for each estrogen`s fecal release over the course of a pregnancy that results in the outcome of birth. As a last step, the estimated urinary and fecal levels were summed to arrive at the cumulative levels of natural estrogens excreted by pregnant women who end up giving birth.

**(k)** Estimated in similar manner with the same data sources as in footnote **(j)**, with the only difference being the gestational age over which the developed excretions curves for the various natural estrogens were integrated. Specifically, the time to an induced abortion, spontaneous abortion and ectopic pregnancy were modeled based on the data of Pazol et al.^13^, Jones and Kost^14^ and Goldhaber and Fireman^41^, respectively.

**(l)** Estimating using data reported by Ventura et al.^7^ and Hoover et al.^40^.

**(m)** Estimated from demographic data as follows: [Women between the average age of menarche (i.e., 12.5 years^1^) and the average age of menopause (i.e., 51 years^6^)]- [Number of menstruating women on OC^5^] - [Number of pregnancies^7^ x (time to birth^41^/5)] - [Number of induced abortions^7^ x (time to induced abortion^13^/52)] - [Number of spontaneous abortions^7^ x (time to spontaneous abortion^14^/52)] - [Number of ectopic pregnancies^(item l)^ x (time to ectopic pregnancy^41^/52)]

**(n)** Excretion per pregnancy outcome (mg/events) × Pregnancy events per year (events/yr) × 1/10^6^ (kg/mg).

**(o)** Estimated as follows:

| **HRT formulations** | **Mass Sold Nationally**  **(2007-2008)^4,17^** | | **Fraction converted to each steroidal estrogen** | **E_1_** | **E_2_** | **E_3_** |
| --- | --- | --- | --- | --- | --- | --- |
|  | Kg/yr |  | | Kg/yr | | |
| Estradiol | 508.6 | 30% (E_1_), 10% (E_2_), 6% (E_3_)^15^ | | 153 | 51 | 31 |
| Conjugated Estrogen | 536.7 | ≈11% (E_1_), ≈2% (E_2_)^31^ | | 59 | 11 |  |
| Esterified Estrogen | 93.8 | ≈15%(E_1_), ≈ 3% (E_2_)  (Estimated from Johnson et al.^31^) | | 14 | 3 |  |
| Estropipate | 36.4 | ≈16%(E_1_), ≈3% (E_2_)  (Estimated from Johnson et al.^31^) | | 5 | 1 |  |
| Estriol | 7.2 | Assumed to be 100% (E_3_), since it is minimally metabolized in the body^33^ | |  |  | 7 |
| Estrone | 1.1 | 17.8% (E_1_), 4.3% (E_2_), < 1% (E_3_)-(Disposition of I.V. Estrone)^32^ | | 0.2 | 0.05 | ≈ 0 |
|  |  | Sum | | **232** | **67** | **38** |

**(p)** Boys less than the age of 12.2 years^16^.

**(q)** Men greater than the age of 12.2 years.

**(r)** Estimated by Anderson et al.^4^ by extending the model of Johnson and Williams^28^.

**(s)** 40 day post-release load, where 40 days is the typical residence time of wastewater in rivers^37^.

**(t)** Estimated as follows E_2–Eq_ = [E_1_]/3·(1 – (f_s_·R_s-E1_+f_ad_·R_ad-E1_))+ [E_2_] ·(1 – (f_s_·R_s-E2_+f_ad_·R_ad-E2_))+ [E_3_]/25·(1 – (f_s_·R_s-E3_+f_ad_·R_ad-E3_)) + 10[EE_2_] ·(1 – (f_s_·R_s-EE2_+f_ad_·R_ad-EE2_). Where: [E_2-Eq_], [E_1_], [E_2_], [E_3_] and [EE_2_] are the mass loads of estradiol equivalents, estrone, estradiol, estriol and ethyinlestradiol, respectively; *f_s_* and *f_a_* are the fractions of wastewater volume that currently undergo secondary and advanced treatment in USA^36^, respectively; R_S-E1_, R_S-E2_, R_S-E3_ and R_S-EE2_ are the removals of E_1_, E_2_, E_3_ and EE_2_ in secondary treatment plants^17^, respectively; R_a-E1_, R_a-E2_, R_a-E3_ and R_a-EE2_ are the removals of E_1_, E_2_, E_3_ and EE_2_ in advanced treatment plants^17^, respectively.

**(u)** Estimated as follows E_2–Eq_ = [E_1_]/3·(1 – (f_s_·R_s-E1_+f_ad_·R_ad-E1_))·(e^-k-E1· tr^)+ [E_2_] ·(1 – (f_s_·R_s-E2_+f_ad_·R_ad-E2_)) ·(e^-k-E2· tr^) + [E_3_]/25·(1 – (f_s_·R_s-E3_+f_ad_·R_ad-E3_)) ·(e^-k-E3· tr^) + 10[EE_2_] ·(1 – (f_s_·R_s-EE2_+f_ad_·R_ad-EE2_) ·(e^-k-EE2· tr^). Where: k-E1, k-E2, k-E3, k-EE2 are first order degradation constants for E_1_, E_2_, E_3_ and EE_2_^17^, respectively; tr is the typical residence time of wastewater in rivers^37^ and all other variables are as previously defined in item (t).
