# Supplementary material for: Contraceptive Options and Their Associated Estrogenic Environmental Loads: Relationships and Trade-Offs
Source: PLoS One. 2014 Mar 26;9(3):e92630. doi: 10.1371/journal.pone.0092630 (PMC3966801; doi:10.1371/journal.pone.0092630)
Supplement: File S2 — Eco-toxicological Potency of Steroidal Estrogens. (DOC) [file pone.0092630.s002.doc]

# S2 Eco-toxicological Potency of Steroidal Estrogens

Based on a critical review of available eco-toxicological data, the European Union has proposed Environmental Quality Standard (EQS) for E_2_ and EE_2_ of 400 and 35 pg/L, respectively.^42,43^ In developing these standards, the committee considered the relative *in vivo* potency of EE_2_ to E_2_ in fish and was of the opinion that the current data suggested that EE_2_ was about 10 times more potent than E_2_.^43^ The Commission’s proposed benchmarks, as well as the relative potency of E_2_ to that of EE_2_, are at odds with the values proposed by an effort led by the pharmaceutical industry.^44^ Specifically, Caldwell et al.^44^ proposed benchmarks of 2,000 and 100 pg/L, respectively, for E_2_ and EE_2_, which puts the relative potency of E_2_ at one-twentieth of that of EE_2_. As detailed in the SCHER opinions^42,43^, the EU regulators do not agree with these industry-derived benchmarks. Hence, for our evaluation, EE_2_ was assumed to be 10 times more potent than E_2_. E_1_ is assumed to be one-third as potent as E_2_.^46^ Limited data has been reported for the *in vivo* eco-toxicological potency of E_3_, however, aquaculture literature suggests that E_3_ is 25 times less potent than E_2_ in inducing sex reversal in fish.^57,58^ Therefore, E_3_ was assumed to be 25 times less potent than E_2_.
