# Supplementary material for: Contraceptive Options and Their Associated Estrogenic Environmental Loads: Relationships and Trade-Offs
Source: PLoS One. 2014 Mar 26;9(3):e92630. doi: 10.1371/journal.pone.0092630 (PMC3966801; doi:10.1371/journal.pone.0092630)
Supplement: File S3 — Modeling the Steroidal Estrogen Load Released Directly Via the Use of a Particular Contraceptive Option (Jd,n). (DOC) [file pone.0092630.s003.doc]

# S3 Modeling the Steroidal Estrogen Load Released Directly Via the Use of a Particular Contraceptive Option (J_d,n_)

*The definitions of all variables used below along with their estimated values are provided in Section S9.*

The steroidal estrogen load released directly via the use of a contraceptive option (*J_d,n_*) is modeled as follows:

$$\text{J}_{\text{d,n}}=\sum_{\text{i}=1}^{\text{I}} \text{L}_{\text{n}}\cdot\text{(U}{}_{\text{i,n}}+\text{F}{}_{\text{i,n}}\text{)}\cdot\frac{\text{Mw}_{\text{i}}}{\text{Mw}_{\text{n}}}\text{.P}_{\text{i}}\cdot365$$

(S1)

where: *i* refers to one of the following estrogens: E_1_, E_2_, E_3_ and EE_2_; *L_n_* is the per user consumption of a given contraceptive option *n* ; *U*_i,n_+ *F*_i,n_ is the net fraction of an administered dose of contraceptive option *n* that is excreted as unchanged and conjugated *i*; *P_i_* is the potency of estrogen *i* relative to that of E_2_; and *Mw_i_* and *Mw_n_* are the molecular weights of *i* and *n,* respectively.

Of the various contraceptive options considered here, the contribution *J_d,n_* is non-zero for EE_2_-based preparations and the recently authorized E_2_-based oral contraceptives only. Parameterization for *L_EE2-OC_* and *L_E2-OC_*, and *U_EE2,EE2_+ F_EE2,EE2_* (i.e., the fractions of an administered dose of EE_2_ excreted in its unchanged form and its conjugated forms) are discussed and estimated below.

**S3.1 Daily Consumption of EE_2_ by Users of EE_2_-based Oral Contraceptives (L_EE2-OC_)**

Two lines of evidence were used to estimate the mean daily per user consumption of EE_2_ (*L_EE2-OC_*). The first approach used to estimate L_EE2_ was based on the mass of EE_2_ used as reported in IMS Health sales data (for the period from March 2007 to February 2008) divided by the estimated number of oral contraceptive users in the USA around the time of the available sales data^5^, as follows:

$\text{L}_{\text{EE}_{2}-\text{OC}}=\frac{\text{82.4 kg/yr (Caldwell et al.}^{17}\text{)}}{\text{10,702,000 } \text{(Mosher and Jones}^{5}\text{)}}\times\frac{10^{9}\text{μg}}{1 \text{kg}}\times\frac{\text{1yr}}{365 \text{d}}=21.1\frac{\text{μg}}{\text{user}\cdot\text{d}}$

The second approach was based on the NSFG survey data reported on the types and proportions with which each EE_2_-based oral contraceptive brand is used within the USA. As described in Table S2 below, this data^38^ was analyzed to arrive at a second independent estimate for L_EE2-OC_ of 22.3 µg of EE_2_/user·d.

As can be seen, the two independent estimates of L_EE2-OC_ yielded remarkably similar values and, hence, the mean of the two estimates, 21.7 µg of EE_2_/user·d, was used for the analyses presented in this study.

**Table S2** Estimate of L_EE2_ Using NSFG Survey Data from Hall and Trussell^38^.

| **Brand^(a)^** | **EE_2_ dose in each**  **active pill^(b)^** | **Correction for inert pills**  **or days of non-use^(b)^** | **% of all OC users^(a)^** | **Daily contribution from the users**  **of each brand ^(c)^** |
| --- | --- | --- | --- | --- |
|  | µg/active pill |  |  | µg/user·d |
| Ortho Tri-Cyclen Lo® | 25 | 0.75 | 12.3 | 2.30 |
| Yasmin® | 30 | 0.75 | 10.8 | 2.43 |
| Yaz® | 20 | 0.86 | 5.6 | 0.97 |
| Ortho Tri-Cyclen® | 35 | 0.75 | 5.2 | 1.37 |
| Tri-Sprintec® | 35 | 0.75 | 4.6 | 1.20 |
| Loestrin FE 1/20® | 20 | 0.75 | 4.7 | 0.71 |
| Trinessa® | 35 | 0.75 | 3.8 | 1.01 |
| Ortho-Cyclen® | 35 | 0.75 | 3.3 | 0.87 |
| Microgestin 1.5/30® | 30 | 0.75 | 3.1 | 0.70 |
| Aviane® | 20 | 0.75 | 2.9 | 0.44 |
| Lutera™ | 20 | 0.75 | 2.5 | 0.38 |
| Seasonale® | 30 | 0.92 | 2.5 | 0.69 |
| Apri® | 30 | 0.75 | 2.4 | 0.54 |
| Sprintec® | 35 | 0.75 | 2.3 | 0.61 |
| Lo/Ovral® | 30 | 0.75 | 1.9 | 0.44 |
| Kariva® | 20 | 0.82 | 1.7 | 0.28 |
| Estrostep FE® | 28.3 | 0.75 | 1.7 | 0.36 |
| Necon 1/35® | 35 | 0.75 | 1.6 | 0.43 |
| Levora® | 30 | 0.75 | 1.5 | 0.34 |
| Trivora® | 32.3 | 0.75 | 1.5 | 0.36 |
| Ortho-Novum 7/7/7® | 35 | 0.75 | 1.3 | 0.35 |
| Ortho-Novum 1/35® | 35 | 0.75 | 1.2 | 0.32 |
| Alesse® | 20 | 0.75 | 1.1 | 0.17 |
| Desogen® | 30 | 0.75 | 1.1 | 0.25 |
| Ortho-Cept® | 30 | 0.75 | 1.1 | 0.25 |
| Mononesessa® | 35 | 0.75 | 1.1 | 0.28 |
| Seasonique® | 30 | 0.92 | 0.9 | 0.26 |
| Zovia 1/50® | 50 | 0.75 | 0.8 | 0.32 |
| Junel FE 20™ | 20 | 0.75 | 0.8 | 0.12 |
| Loestrin 21 1.5/30® | 30 | 0.75 | 0.8 | 0.18 |
| Mircette® | 18.1 | 0.93 | 0.7 | 0.11 |
| Cryselle® | 30 | 0.75 | 0.6 | 0.14 |
| Ovcon 35® | 35 | 0.75 | 0.6 | 0.17 |
| Levlen® | 30 | 0.75 | 0.6 | 0.13 |
| Nortrel 28® | 35 | 0.75 | 0.6 | 0.15 |
| Oegstrel® | 50 | 0.75 | 0.6 | 0.22 |
| Triphasil® | 32.4 | 0.75 | 0.5 | 0.13 |
| Tri-Levlen® | 32.4 | 0.75 | 0.5 | 0.13 |
| Necon 7/7/7® | 35 | 0.75 | 0.5 | 0.12 |
| Low-Ogestel® | 30 | 0.75 | 0.5 | 0.11 |
| Cyclessa® | 25 | 0.75 | 0.5 | 0.09 |
| Enpresse® | 32.4 | 0.75 | 0.5 | 0.12 |
| Portia® | 30 | 0.75 | 0.5 | 0.11 |
| Nortrel 7/7/7® | 35 | 0.75 | 0.4 | 0.10 |
| Femcon FE™ | 35 | 0.75 | 0.4 | 0.10 |
| Ortho-Novum 1/50® ^(d)^ | [50](http://www.sciencedirect.com/science/article/pii/S0010782412002661#tf0020) | 0.75 | 0.3 | 0.12 |
| Zovia 1/35E® | 35 | 0.75 | 0.3 | 0.08 |
| Nordette® | 30 | 0.75 | 0.3 | 0.06 |
| Norethin 1/35E | 35 | 0.75 | 0.3 | 0.07 |
| Loestrin 24FE® | 20 | 0.86 | 0.3 | 0.05 |
| Jenest® | 35 | 0.75 | 0.3 | 0.07 |
| Ocella™ | 30 | 0.75 | 0.2 | 0.05 |
| Levlite™ | 20 | 0.75 | 0.2 | 0.03 |
| Nortrel 0.5/0.035® | 35 | 0.75 | 0.2 | 0.06 |
| Ovcon 50® | 50 | 0.75 | 0.2 | 0.08 |
| Reclipsen™ | 30 | 0.75 | 0.2 | 0.05 |
| Necon 1/50® **^(d)^** | [50](http://www.sciencedirect.com/science/article/pii/S0010782412002661#tf0020) | 0.75 | 0.2 | 0.08 |
| Demulen 1/35® | 35 | 0.75 | 0.2 | 0.04 |
| Modicon® | 35 | 0.75 | 0.2 | 0.04 |
| [Ortho-Novumd®](http://www.sciencedirect.com/science/article/pii/S0010782412002661#tf0025) | 35 | 0.75 | 0.2 | 0.04 |
| Ortho-Novum 10/11® | 35 | 0.75 | 0.2 | 0.04 |
| Kelnor™ | 35 | 0.75 | 0.2 | 0.04 |
| Velivet™ | 25 | 0.75 | 0.2 | 0.03 |
| Jolessa™ | 30 | 0.92 | 0.1 | 0.03 |
| Demulen 1/50® | 50 | 0.75 | 0.1 | 0.04 |
| Junel 30™ | 30 | 0.75 | 0.1 | 0.02 |
| Tri-Previfem™ | 35 | 0.75 | 0.1 | 0.03 |
| Junel FE 30™ | 30 | 0.75 | 0.1 | 0.02 |
| Norinyl 1+35® | 35 | 0.75 | 0.1 | 0.03 |
| Ovral® | 50 | 0.75 | 0.1 | 0.04 |
| Nortrel® | 35 | 0.75 | 0.1 | 0.03 |
| Lessina® | 20 | 0.75 | 0.1 | 0.02 |
| Zenchent® | 35 | 0.75 | 0.1 | 0.03 |
| [Zovia®d](http://www.sciencedirect.com/science/article/pii/S0010782412002661#tf0025) | 35-50 | 0.75 | 0.1 | 0.01-0.02 |
| Microgestin FE 1/20® | 20 | 0.75 | 0.1 | 0.01 |
| Previfem™ | 35 | 0.75 | 0.1 | 0.01 |
| Necon 10/11® | 35 | 0.75 | 0.1 | 0.01 |
| Norinyl 1/50® ^(d)^ | [50](http://www.sciencedirect.com/science/article/pii/S0010782412002661#tf0020) | 0.75 | 0.1 | 0.02 |
| Junel 20™ | 20 | 0.75 | 0.1 | 0.01 |
| Balziva™ | 35 | 0.75 | 0.1 | 0.01 |
| Azurette™ | 20 | 0.75 | 0.1 | 0.01 |
| Mercilon® | 20 | 0.82 | 0.1 | 0.01 |
| [Miranovae](http://www.sciencedirect.com/science/article/pii/S0010782412002661#tf0030) | 20 | 0.75 | 0.1 | 0.01 |
| Nueva Perla | 30 | 0.75 | 0.1 | 0.02 |
| [Microgynone](http://www.sciencedirect.com/science/article/pii/S0010782412002661#tf0030) | 30 | 0.75 | 0.1 | 0.01 |
| Valette | 30 | 0.75 | 0.1 | 0.01 |
| [Nocicline](http://www.sciencedirect.com/science/article/pii/S0010782412002661#tf0030) | 30 | 0.75 | 0.1 | 0.01 |
| Gynera | 30 | 0.75 | 0.1 | 0.01 |
|  |  |  | **L_EE2-OC_** | **22.3** |

**Foonotes:** (a) From Hall and Trusell^38^; (b) From Hall and Trusell^38^ and/or respective drug monographs^48^; (c) Estimated for each brand as follows: EE_2_ dose in each active pill × correction for inert pills × % of all OC users/100; (d) Not included in the summation since these are Mestranol based brands

**S3.2 Daily Consumption of E_2_ by Users of E_2_-based Oral Contraceptives (L_E2_)**

Over the 28-day dosing regimen of the recently authorized E_2_-based oral contraceptive, Natazia, its users end up administering 52 mg of estradiol valerate^39^. Hence, on an average daily basis, the users of Natazia administer 1.86 mg of estradiol valerate, which is equivalent to 1.42 mg of free estradiol.

**S3.3 Fraction of Administered EE_2_ Excreted Unchanged and as Conjugates via Urinary and Fecal Excretions (U_EE2,EE2_+ F_EE2,EE2_)**

Overall, the analysis presented below of the excretion profile of EE_2_ was done to assess the suitability of the assumption that 50% of EE_2_ dose is released in unchanged form and as it glucuronide and sulphate conjugates^4,17^.

The urinary excretion profile of EE_2_ is summarized in Table S3 below. Urinary elimination of EE_2_ is largely complete 5 days post-administration.^19,20^ The pooled data of the number of subjects from all studies in which the elimination of EE_2_ was monitored for the minimum of five days^18-20,22,23^ suggests that 34% (n=38) of an administered dose EE_2_ is eliminated via the urinary route. As initially noted by Johnson and Williams^28^, considerable uncertainty exists as to the fraction of the net urinary levels that is in the form of unchanged EE_2_ and its conjugates. Over time, the fraction of the urinary excretions that is composed of EE_2_ and its conjugates decreases.^19,20^ Recognizing this, only the data of Reed^20^ and Abdel-Aziz and Williams^19^ was pooled and weighted by the number of subjects to estimate that approximately 40% of the total urinary levels are present as free EE_2_ and its glucuronide conjugates. The sulfate conjugates of EE_2_ at best amounts to an additional 2% of the net urinary release.^21,27^ Therefore, 42% of the net urinary levels is estimated to be present as free EE_2_ and its conjugates. Multiplying this by the net level of EE_2_ expected to be eliminated via the urinary route of 34% (estimated above) allows one to estimate that 14.3% administered dose of EE_2_ is eliminated via the urinary route as free EE_2_ and its conjugates.

**Table S3** Urinary Excretion Profile of Ethyinlestradiol.

| **Ref.** | **n** | **Duration** | **Dose** | **Total** | | | | | |  | **EE_2_** | | | | |
| --- | --- | --- | --- | --- | --- | --- | --- | --- | --- | --- | --- | --- | --- | --- | --- |
|  |  |  |  | **Total** | **Free** | **Glucuronide** | | | **Sulphate** |  | **Total** | | **Free** | **Glucuronide** | **Sulphate** |
|  |  | **hr** | **µg** | **% Dose** | **% Urine radioactivity** | | | | |  | **% Dose** | | **% Urine radioactivity** | | |
| **(18)** | 5 | 120 | 50 | 28 ± 3.4 | 16.7 | 74 | | | 10 |  |  | |  |  |  |
| **(19)** | 9 | 120 | 64 | 40.5 ± 8.5 |  |  | | | |  |  | |  |  |  |
|  | 2 | 120 | 64 |  | < 1 | 67 | | |  |  |  | |  | 12.4,19.4 |  |
|  | 2 | 21.5 | 64 |  | < 1 |  | | |  |  |  | |  | 31,45 |  |
| **(20)** | 13 | 120 | 50 | 30.4 ± 1.5 | 18.8 | 65.4 | | | 10.9 |  |  | | 20 | 23 |  |
| **(21)** | 4-9 | 48-72 | 25 |  | 3.8 | 70.4 | | | 11.6 |  |  | |  |  | <1.8 |
| **(22)** | 3 | 120 | 25 | 31 ± 4.3 | <3 |  | | | |  |  | |  |  |  |
| **(23)** | 8 | 240 | 50 | 38 ± 8 |  |  | | | |  |  | |  |  |  |
| **(24)** | 6 | 72 | 50 | 27 ± 11 | 3 | 68 | | 16 | |  |  | |  |  |  |
| **(25)** | 6 | 72 | 50 | 17 ± 8 | 1-10 | 66-95 | | | |  |  | |  |  |  |
| **(26)** | 6 | 48 | 50 | 24.6 | 9 | 76 | | | |  |  | |  |  |  |
|  | 6 | 24 | 50 | 17 |  |  | | | |  |  | |  | 31 | |
| **(27)** | 4 | 24 | 50 | 9 | 6 | 68 | 15 | | |  |  | |  | 37.4 | 2 |
|  | 4 | 72 | 50 | 18.1 |  |  | | | |  |  | |  |  |  |
|  |  |  |  |  |  |  | | | |  | |  |  |  |  |

Fecal elimination of EE_2_ has only been reported in a handful of studies.^20,23,29^ Of these, the most comprehensive was that of Speck et al.^23^ who, upon studying fecal elimination for 10 days in 8 subjects, demonstrated that approximately 62% of the administered dose of EE_2_ was eliminated via this route. Unfortunately, this study did not report on the fraction of the net fecal release that was present in the form of unchanged EE_2_. However, Reed et al.^20^, upon the analysis of fecal collections from a single male subject, suggested that 30% was eliminated via the fecal route in the form of EE_2_. Further, it has been suggested that the fecal elimination of EE_2_ largely results from biliary excretions.^20,29^ Maggs et al.^25^ reported that on average 37% (n=5) of biliary excretions were in the form of EE_2_ conjugates, which is consistent with the values measured by Reed et al. for the release of EE_2_ in a single fecal sample. Overall, in the present study, the fecal elimination was estimated by multiplying the fraction of biliary excretions that is present as conjugated EE_2_^25^ by the net levels of EE_2_ eliminated via the fecal route^23^ to arrive at an estimate of 22.9%.

By summing the fecal and urinary elimination levels estimated above, it is estimated that 37.2% of an administered dose of EE_2_ is eliminated as EE_2_ and its conjugates.
