# Supplementary material for: Contraceptive Options and Their Associated Estrogenic Environmental Loads: Relationships and Trade-Offs
Source: PLoS One. 2014 Mar 26;9(3):e92630. doi: 10.1371/journal.pone.0092630 (PMC3966801; doi:10.1371/journal.pone.0092630)
Supplement: File S4 — Modeling the Load of Natural Estrogens Released over the Course of Unintended Pregnancies that can be Associated with a User's Choice to Use a Particular Contraceptive Option (Jf,n). (DOC) [file pone.0092630.s004.doc]

# S4 Modeling the Load of Natural Estrogens Released over the Course of Unintended Pregnancies that can be Associated with a User’s Choice to Use a Particular Contraceptive Option (J_f,n_)

*The definitions of all variables used below along with their estimated values are provided in Section S9.*

The total load of natural steroidal estrogens released over the course of unintended pregnancies that can be associated with a user’s specific choice of a given contraceptive option is estimated as follows:

$$\text{J}_{\text{f,n}}= \sum_{\text{j}=1}^{\text{J}} \frac{\text{A}_{\text{n}}}{100}\cdot\left(\text{f}_{\text{a,n}}\int_{\text{t}=0}^{\text{t}_{\text{a}}} \left(\text{U}_{\text{j}}\text{(t)}+\text{F}_{\text{j}}\text{(t)}\right)\text{dt}+\text{f}_{\text{b,n}}\cdot\text{f}_{\text{u}}\int_{\text{t}=0}^{\text{t}_{\text{b}}} \left(\text{U}_{\text{j}}\text{(t)}+\text{F}_{\text{j}}\text{(t)}\right)\text{dt}+\text{f}_{\text{m,n}}\int_{\text{t}=0}^{\text{t}_{\text{m}}} \left(\text{U}_{\text{j}}\text{(t)}+\text{F}_{\text{j}}\text{(t)}\right)\text{dt}+\text{f}_{\text{e,n}}\int_{\text{t}=0}^{\text{t}_{\text{e}}} \left(\text{U}_{\text{j}}\text{(t)}+\text{F}_{\text{j}}\text{(t)}\right)\text{dt}\right)\cdot\text{P}_{\text{j}}$$

(S2)

In the equation above, *j* refers to the individual natural steroidal estrogens E_1_, E_2_, and E_3_. *A_n_* is the annual probability that a typical user for a given contraception option *n* will experience an unintended pregnancy during the first year of use due to contraception failure^51^. *f_a,n_*, *f_b,n_*, *f_m,n_, f_e,n_* are the fractions of an unintended pregnancy experienced by the users of contraceptive option *n* that will result in the outcomes of induced abortion, birth, spontaneous abortion and ectopic pregnancy^52^, respectively. *f_u_* is the fraction of all unintended births that are unwanted. Such a correction/adjustment factor is not applied to all other outcomes of unintended pregnancies since these outcomes do not end up satisfying a future need of the parents for a child. *t_a_*, *t_b_*, *t_m_* and *t_e_* are the durations of unintended pregnancies that result in the outcomes of induced abortion, birth, spontaneous abortion and ectopic pregnancy, respectively.^13,14,41^

*U_j_(t)* and *F_j_(t)* are the gestation age-dependent releases of natural estrogens via the urinary and fecal excretions of pregnant women, respectively. The urinary excretions curves for each estrogen *j* as function of gestational age, *U_j_*(t), were estimated by multiplying the data reported by Berg and Kuss^8^ for the excretion curves of natural estrogens for women of various gestational ages expressed in units of grams of estrogen per gram of creatinine by the average daily creatinine excretion for pregnant women (g of creatinine/24hr)^9^. To date, only a handful of analyses have been performed for the fecal release of natural estrogens by pregnant women.^9,12^ Therefore, as a best approximation, the limited fecal release data available^11,12^ were used to estimate fecal-to-urinary excretion ratios for each natural estrogen. Subsequently, these estimated ratios were multiplied by their respective *U_j_*(t) values to arrive at best possible estimates for *F_j_*(t). *P_j_* is the potency of estrogen *j* relative to that of E_2_ (see Section S2).
