# Supplementary material for: Contraceptive Options and Their Associated Estrogenic Environmental Loads: Relationships and Trade-Offs
Source: PLoS One. 2014 Mar 26;9(3):e92630. doi: 10.1371/journal.pone.0092630 (PMC3966801; doi:10.1371/journal.pone.0092630)
Supplement: File S5 — Modeling the Legacy Estrogenic Load Arising from an Unwanted Birth and can be Associated with a User's Choice to Use a Particular Contraceptive Option (JL,n). (DOC) [file pone.0092630.s005.doc]

# S5 Modeling the Legacy Estrogenic Load Arising from an Unwanted Birth and can be Associated with a User’s Choice to Use a Particular Contraceptive Option (J_L,n_)

*The definitions of all variables used below along with their estimated values are provided in Section S9.*

The legacy estrogen load resulting from an unwanted birth that is manifested over the life span of the resulting child and the duration of its genetic lineage (i.e., that child’s progeny) is expressed as follows:

$\text{J}_{\text{L,n}}= \frac{\text{A}_{\text{n}}}{100}\cdot\text{f}_{\text{b}}\cdot\text{f}_{\text{u}}\cdot\text{M}_{\text{L}}$ (S3)

In Eq. (S3), M_L_ is the estrogenic legacy load of an unwanted birth and its genetic lineage. This is an inherently difficult parameter to quantify with certainty. For example, consider the work of Murtaugh and Schlax^55^ in which the medium variant fertility projections from the World Population Prospects’ 2006 revision^50^ were used to estimate that the duration of a single birth’s average genetic legacy in the USA is 470 years, which is nearly six times the average life span of a person in the USA.^49^ Any attempt at quantifying *M_L_* would not only require that one be able to predict all contraceptive options that will be available over such a lengthy duration but also requires the one can establish the mix with which future populations choose to use these available options. As such, we purposefully do not attempt to use this approach to estimate *M_L_* here because we believe any such attempt is likely to be presumptive. However, a defensible estimate for *M_L_* can be made using a highly conservative approach as described by the following expression:

$$\text{M}_{\text{L}}= \text{G}_{\text{L}}\cdot\left(\left(\text{L}_{\text{BF}}+\text{L}_{\text{FT}}\right)\cdot\text{SR}_{\text{F}}+\text{L}_{\text{BM}}\cdot\text{SR}_{\text{M}}\right)$$

(S3a)

In this expression, *G_L_* is the duration of a single birth’s genetic legacy in the USA, for which Murtaugh and Schlax’s estimate of 470 years^55^ is adopted here. *L_BF_* and *L_BM_* are the life-span averaged loads of natural estrogens basally excreted by females and males, respectively, and are estimated below in section 5.1 and 5.3 respectively. These loads only account for the release of natural estrogens from those anthropogenic excretions that a will always be present (i.e., the basal levels excreted as the females and males progress through their various life stages). *L_FT_* is the life-span averaged annual load of natural estrogens due to an assumed future total fertility rate (TFR) of 1.85 and is estimated below in section 5.2. The latter TFR of 1.85 was assumed as the basis for an estimate of *L_FT_* for several reasons: (1) this estimate for the future fertility rate largely served as the basis for Murtaugh and Schlax’s estimate for *G_L_*; and (2) it is considered to be a conservative estimate for future fertility rates in the USA.^59, 60^ The latter assertion is justified below. SR_f_ and SR_m_ are the respective fractions of births in the USA that are females and males, respectively, and are estimated using the at-birth sex ratio currently observed in that country.^56^ The parameterization of variables in Eq. (S3a) yields an estimate of M_L_ of 2.1 grams of E_2-eq_ per unwanted birth.

The estimate for M_L_ that the above parameterization yields is highly conservative (i.e., it will result in an underestimate of legacy load) and, hence, is defensible for a number of reasons. Firstly, the uncertain contribution due to the genetic legacy’s use of future contraceptive options and HRT preparations is not included. Also not included, for similar reasons, is the genetic legacy’s use of other future estrogenic chemicals. Lastly, both the parameterization for *G_L_* and *L_FT_* are likely conservative estimates since more recent future fertility projection developed for the United Nation’s World Population Prospects’ 2010 revision suggest a medium variant TFR of 2.1 for the USA^59,60^. This projected level of 2.1 is equal to replacement level fertility and as long as such a fertility rate is maintained within a population the value of *G_L_*, and hence *M_L_*, would not converge to a finite value.

**S5.1 Life-span-averaged Basal Estrogen Load of a Female in the USA (L_BF_)**

In the USA, the average female will be pre-pubescent for 12.5 years^1^, menstruate for another 38.8 years^1,6^ and be menopausal for 29.6 years^6,49^ before death at the age of 80.6 years^49^. As shown in Table S4 below, such a life history can be used to estimate that a female basally releases a minimum of 124 mg of E_2_-eq over her life span to sewers. Given that the average life span of a female is 80.6 years^49^, the life-span-averaged basal estrogen load (*L_BF_*) for a female in the USA can be estimated to amount to 1.54 mg of E_2_-eq/yr (i.e., *L_BF_*).

**Table S4** Estimation of the Life-span Basal Estrogen Load of a Female in the USA.

| Life stage | | Duration of life stage | Daily per capita excretion during the life stage^(a)^ | | | | | Life-span load^(b)^ |
| --- | --- | --- | --- | --- | --- | --- | --- | --- |
|  |  |  | E_1_ | | E_2_ | | E_3_ | E_2-Eq_ |
|  |  | yr | µg/cap·d | | | | | mg |
| Prepubescent girls | | 12.5 | 0.08 | | 0.06 | | 0.2 | 0.43 |
| Menstruating women | | 38.5 | 8.69 | | 4.42 | | 9.4 | 108 |
| Menopausal women | | 29.6 | 1.7 | | 0.79 | | 1.9 | 15.5 |
|  | |  |  | |  | |  | 124 |
|  |  | |  |  | |  | |  |

**Footnotes:** (a) See Table S1; (b) E_2–Eq_ = [E_1_]/3 + [E_2_] + [E_3_]/25 where: [E_2-Eq_], [E_1_], [E_2_] and [E_3_] are the mass loads of estradiol equivalents, estrone, estradiol and estriol, respectively. The mass loads for all life stages were estimated as follows: daily per capita excretion (µg/cap·d) × duration of life stage (yr) × 365 (d/yr) × 1/10^3^ (mg/µg).

**S5.2 Life-span-averaged Estrogen Load of a Female in the USA due to Total Fertility** (**L_FT_)**

In the present study, as described above, the future fertility rate of a woman in the USA is assumed to be 1.85 (i.e., 1.85 live births over her lifetime), which is the United Nations’ 2006 mid-variant estimate for the level to which total fertility rate was projected to converge in the USA by 2050^50^. Pregnancy outcomes other than births were not considered since future trends for these outcomes are difficult to predict. Nevertheless, pregnancies that result in outcomes other than birth are comparatively minor contributors of estrogenic content (see Table S1). For example, consider that a pregnancy resulting in a birth leads to the release of an E_2_-eq load that is 20, 25 and 32 times higher than the levels released had the pregnancy instead resulted in the outcome of spontaneous abortion, induced abortion and ectopic pregnancy, respectively.

The analysis summarized in Table S5 suggests that, with an assumed fertility rate of 1.85, a female would release 520 mg of E_2_-eq to sewer systems over her lifetime. As noted earlier, the average life span of a female in the USA is 80.6 years^49^. This estimate, along with the estimates from Table S6 below, suggest that the life-span-averaged estrogen load due to the fertility of a female in the USA can be estimated to be 6.5 mg of E_2_-eq/yr (i.e., *L_FT_*).

**Table S5** Estimation of the Life-Span Estrogen Fertility Load for a Female with a Fertility Rate of 1.85.

| Events | Fertility rate | Excretion/birth^(a)^ | | | Life-span load^(b)^ |
| --- | --- | --- | --- | --- | --- |
|  |  | E_1_ | E_2_ | E_3_ | E_2-Eq_ |
|  | Births/women | mg/birth | | | mg |
| Pregnancies  resulting in births | 1.85 | 259 | 134 | 1525 | 520 |

**Footnotes:** (a) See Table S1; (b) E_2–Eq_ = [E_1_]/3 + [E_2_] + [E_3_]/25 where [E_2-Eq_], [E_1_], [E_2_] and [E_3_] are the mass loads of estradiol equivalents, estrone, estradiol and estriol, respectively. The mass loads were estimated as follows: fertility rate (Births/Life) × excretion per birth (mg/birth).

**S5.3 Life-span-averaged Basal Estrogen Load of a Male in the USA (L_BM_)**

The average male in the USA will be pre-pubescent for 12.2 years^16^. For the remainder of his life, it is assumed that he will excrete as an adult male before dying at the age of 75.6 years^49^. This data can be used to estimate the minimum life-span averaged annual load of E_2_-eq of a male in the USA. Specifically, the analysis in Table S6 below suggests that an American male basally releases a minimum of 79.7 mg of E_2_-eq over his life span to sewers. Noting that the average life span of an American male is 75.6 years^49^, the life-span averaged basal estrogen load for a male in the USA is estimated to be 1.05 mg of E_2_-eq/yr (i.e., L_BM_).

**Table S6** Estimate of the Life-span Basal Estrogen Load of a Male in the USA.

| Life stages | Duration of life stage | Daily per capita excretion during the life stage^(a)^ | | | Life-span load^(b)^ |
| --- | --- | --- | --- | --- | --- |
|  |  | E_1_ | E_2_ | E_3_ | E_2-Eq_ |
|  | yr | µg/cap·d | | | mg |
| Prepubescent boy | 12.2 | 0.08 | 0.06 | 0.2 | 0.42 |
| Adult Male | 63.4 | 3.6 | 2.1 | 2.4 | 79.3 |
|  |  |  |  | Sum | 79.7 |

**Footnotes:** (a) See Table S1; (b) E_2–Eq_ = [E_1_]/3 + [E_2_] + [E_3_]/25 where [E_2-Eq_], [E_1_], [E_2_] and [E_3_] are the mass loads of estradiol equivalents, estrone, estradiol and estriol, respectively. The mass loads for all life stages were estimated as follows: daily per capita excretion (µg/cap·d) × Duration of life stage (yr) × 365 (d/yr) × 1/10^3^ (mg/µg).
