# Supplementary material for: Contraceptive Options and Their Associated Estrogenic Environmental Loads: Relationships and Trade-Offs
Source: PLoS One. 2014 Mar 26;9(3):e92630. doi: 10.1371/journal.pone.0092630 (PMC3966801; doi:10.1371/journal.pone.0092630)
Supplement: File S6 — Pregnancy Events and Estrogenic Load Averted by a Population's Collective Use of Contraception. (DOC) [file pone.0092630.s006.doc]

# S6 Pregnancy Events and Estrogenic Load Averted by a Population’s Collective Use of Contraception

*The definitions of all variables used below along with their estimated values are detailed in Section S9.*

**S6.1 Estrogenic Load Averted by a Population’s Collective Use of Contraception**

The net estrogenic load averted through the collective use of contraception by a given population was estimated using the following model:

E_a_ = E_p_ + E_L_- E_e_ (S4)

In Eq. (S4), E_a_ is the net estrogenic averted through a population’s collective use of contraception. E_p_ is the estrogenic load that would have been released over the course of all pregnancies averted due to the population’s collective use of contraception. E_L_ is the estrogenic load represented by all unwanted legacies averted through the population’s collective use of contraception. E_e_ is the estrogenic content directly released via the population’s collective use of contraception.

E_p_ was estimated using the following model

$$\text{E}_{\text{p}}= \text{P}_{\text{T}}\cdot\text{(IP}{}_{\text{e}}\text{-IP}_{\text{o}}\text{)}\cdot\left[\sum_{\text{j}=1}^{\text{J}} \text{ P}_{\text{j}}\cdot\left(\text{f}_{\text{a}}\int_{\text{t}=0}^{\text{t}_{\text{a}}} \left(\text{U}_{\text{j}}\text{(t)}+\text{F}_{\text{j}}\text{(t)}\right)\text{ dt}+\text{f}_{\text{b}}\cdot\text{f}_{\text{u}}\int_{\text{t}=0}^{\text{t}_{\text{b}}} \left(\text{U}_{\text{j}}\text{(t)}+\text{F}_{\text{j}}\text{(t)}\right)\text{ dt}+\text{ f}_{\text{m}}\int_{\text{t}=0}^{\text{t}_{\text{m}}} \left(\text{U}_{\text{j}}\text{(t)}+\text{F}_{\text{j}}\text{(t)}\right)\text{ dt}+\text{ f}_{\text{e}}\int_{\text{t}=0}^{\text{t}_{\text{e}}} \left(\text{U}_{\text{j}}\text{(t)}+\text{F}_{\text{j}}\text{(t)}\right)\text{ dt}\right)\text{ }\right]$$

(S4a)

In Eq. (S4a), *P_T_* is the total number of women using any form of contraception.^5^ *IP_o_* is the rate at which *P_T_* currently experiences unintended pregnancies.^53^ *IP_e_* is the unintended pregnancy rate expected to be observed among the user population *P_T_* should they all stop using their respective contraceptive methods. Since the accurate estimation of *IP_e_* represents a significant challenge, it is conservatively estimated here. A conservative estimate for *IP_e_* can be furnished by assuming that it is equal to the level of unintended pregnancies observed among those women who currently do not use any form of contraception and due to their sexual activity are at a risk of experiencing unintended pregnancies.^47,53^ The reason the use of such data is considered to produce a conservative estimate for *IP_e_*, and hence, *E_P_*, is that these sexually active non-users of contraception have been suggested to represent a selected population cohort that experiences lower-than-normal level of sexual activity and fecundity^53^. *f_a_*, *f_b_*, *f_m_* and *f_e_* are overall levels of all pregnancies averted that are taken to the outcome of induced abortion, birth, spontaneous abortion and ectopic pregnancy, respectively.^40,47^ All other variables are as previously stated.

*E_L_* was estimated using the following model:

$\text{E}_{\text{L}}= \text{P}_{\text{T}}\cdot\text{(IP}{}_{\text{e}}\text{-IP}_{\text{o}}\text{)}\cdot\left[\text{f}_{\text{b}}\cdot\text{f}_{\text{u}}\cdot\text{M}{}_{\text{L}}\right]$

(S4b)

All variables in Eq. (S4b) have been previously defined.

*E_e_* was estimated as in section S1 (see footnote f Table S1).

**S6.2 Pregnancy Events Averted by a Population’s Collective Use of Contraception**

The net number of pregnancy events averted through a population’s collective use of contraception was estimated by using the following model:

$$\text{N}_{\text{averted}}= \text{P}_{\text{T}}\cdot\text{(IP}{}_{\text{e}}\text{-IP}_{\text{o}}\text{)}\cdot\left[\text{f}_{\text{a}}+\text{f}_{\text{b}}\cdot\text{f}_{\text{w}}+\text{f}_{\text{m}}+\text{f}_{\text{e}}\right]$$

(S5)

All variables in Eq. (S5) have been previously defined.

The number of each pregnancy outcome averted by a population’s collective use of contraception was estimated using the following simplifications of Eq. (S5):

| Number of abortions  averted | $\text{N}_{\text{a-averted}}\text{ }= \text{P}_{\text{T}}\cdot\text{(IP}{}_{\text{e}}\text{-IP}_{\text{o}}\text{)}\cdot\left[\text{f}_{\text{a}}\right]$ | (S5a) |
| --- | --- | --- |
| Number of unwanted births  averted | $\text{N}_{\text{ub-averted}}\text{ }= \text{P}_{\text{T}}\cdot\text{(IP}{}_{\text{e}}\text{-IP}_{\text{o}}\text{)}\cdot\left[\text{f}_{\text{b}}\cdot\text{f}_{\text{w}}\right]$ | (S5b) |
| Number of miscarriages  averted | $\text{N}_{\text{m-averted}}\text{ }= \text{P}_{\text{T}}\cdot\text{(IP}{}_{\text{e}}\text{-IP}_{\text{o}}\text{)}\cdot\left[\text{f}_{\text{m}}\right]$ | (S5c) |
| Number of ectopic pregnancies averted | $\text{N}_{\text{e-averted}}\text{ }= \text{P}_{\text{T}}\cdot\text{(IP}{}_{\text{e}}\text{-IP}_{\text{o}}\text{)}\cdot\left[\text{f}{}_{\text{e}}\right]$ | (S5d) |
