# Supplementary material for: Contraceptive Options and Their Associated Estrogenic Environmental Loads: Relationships and Trade-Offs
Source: PLoS One. 2014 Mar 26;9(3):e92630. doi: 10.1371/journal.pone.0092630 (PMC3966801; doi:10.1371/journal.pone.0092630)
Supplement: File S7 — Estimating Relative Contributions as Shown in Figure 3 (Main Body). (DOC) [file pone.0092630.s007.doc]

# S7 Estimating Relative Contributions as Shown in Figure 3 (Main Body).

All loads were estimated on a pre-treatment basis.

The number of events for each pregnancy outcome was estimated from Finer and Henshaw^47^, Hoover et al.^40^ and Ventura et al.^7^.

The resultant estrogenic load released over the course of each pregnancy outcome was estimated as follows: number of events for a pregnancy outcome^7, 40, 47^ multiplied by the estrogenic load excreted over course of the pregnancy outcome (See Table S1).

The estrogenic legacy represented by each birth category was estimated as follows: number of births per category^7,47^ multiplied by M_L_ (Eq. (S3a)).
