# Supplementary material for: Contraceptive Options and Their Associated Estrogenic Environmental Loads: Relationships and Trade-Offs
Source: PLoS One. 2014 Mar 26;9(3):e92630. doi: 10.1371/journal.pone.0092630 (PMC3966801; doi:10.1371/journal.pone.0092630)
Supplement: File S8 — Modeling Estrogen Loads Arising from Changes in Contraceptive Use. (DOC) [file pone.0092630.s008.doc]

# S8 Modeling Estrogen Loads Arising from Changes in Contraceptive Use

*The definitions of all variables used below along with their estimates values are provided in Section S9.*

In order to estimate the changes in steroidal estrogen loads that result from changes in contraceptive use, it is necessary to evaluate the estrogen loads for the *status quo* scenario (i.e., the resulting from the mix of contraceptive options currently in use) and compare them to the loads experienced under alternate scenarios.

**S8.1 Modeling Steroidal Estrogens Loads for the *Status Quo* Scenario**

The estrogenic load that can be ascribed to a group of first-year EE_2_-OC users under the *status quo* scenario is estimated using the following equation:

$$\text{E}_{\text{EE2-OC}}= \text{P}_{\text{s}}\cdot\text{[J}_{\text{d,EE2 OC}}+\text{J}_{\text{f,EE2 OC}}+\text{J}_{\text{L,EE2 OC}}\text{]}$$

(S6)

In Eq. (S6), for the purpose of the scenario analysis, *P_s_* is chosen here to be a unit population of 1,000 first year users of EE_2_-OC. *J_d,EE2 OC_*, *J_f,EE2 OC_* and *J_L,EE2 OC_* were estimated using Eq. (S1), (S2) and (S3), respectively.

**S8.2 Modeling Steroidal Estrogens Loads for Scenarios Where Users Switch to Non-Estrogen Based Contraceptive Methods**

Upon discontinuing the use of EE_2_-OC, the switching population, *P_s_*, is expected to either adopt other contraceptive options or discontinue the use of contraception altogether.^45^ Under the *Discontinue EE_2_* scenario, the expected load of steroidal estrogens that can be attributed to contraceptive choices made by *P_s_* can be modeled as follows:

$$\text{E}_{\text{S}}= \text{P}_{\text{s}}\cdot\left[\sum_{\text{m}=1}^{\text{M}} \text{f}_{\text{s, m}}\cdot\text{(J}_{\text{f,m}}+\text{J}_{\text{L,m}}\text{)}+\text{f}_{\text{no}-\text{method}}\cdot\text{(J}_{\text{f,no-method}}+\text{J}_{\text{L,no-method}}\text{)}\right]$$

(S7)

In Eq. (S7), *M* is the total number of individual contraceptive options, *m*, adopted by the *P_s_* after they discontinue the use of EE_2_**-**OC and *f_s,m_* is the fraction with which P_s_ adopts each option *m*. *f_no-method_* is the fraction of *P_s_* that completely abandons the use of contraception after discontinuing the use of the EE_2_**-**OC. Values for *f_s,m_* and *f_no-method_* are estimated using the data of Rosenberg and Waugh^45^ who reported the contraceptive mix adopted by those individuals who for various reasons discontinued the use of EE_2_*-*OC and still wanted to prevent a pregnancy. *J_f,m_* and *J_f,no-method_* were estimated using Eq. (S2) and *J_L,m_* and *J_L,no-method_* using Eq. (S3).

In addition to the most likely switch scenario, we also consider three more explorative scenarios as per the suggestion of Wise et al.^10^ in which the entire switching population, *P_s_*, is modeled as switching from EE_2_-based oral contraceptives to the use of male condoms or diaphragms or a copper IUD. These scenarios are modeled by simplifying the coefficients of Eq. (S7).

**S8.3 Modeling Changes in Flows of Steroidal Estrogens Should EE_2_-OC Users Switch to E_2_-OC**

The pearl index (i.e., number of unintended pregnancies per 100 women-years of contraceptive exposure) for the newly introduced E_2_-OC has been reported to be similar to that of EE_2_-OC.^61^ Perhaps more importantly, since the manner with which the E_2_-OC is administered is exactly the same as EE_2_-OCs (i.e., one pill a day), the typical failure rate of the E_2_-OC is expected to be similar to that of EE_2_-OCs. Therefore, the estrogenic loads *J_f_* and *J_L_* for the two forms OCs can be assumed to be comparable. Further, this suggests that the only difference in terms of estrogenic load associated with these two forms of OC, results the from the estrogenic content that is released directly via the excretion of the respective users (i.e., J_d_). Moreover, it is also important to recognize that, clinically a 2 mg/cap·d dose of estradiol valerate (i.e., the salt form of estradiol used in Natazia) is equivalent to a 20 µg/cap·d dose of ethyinlestradiol.^61^ This, along with the estimates furnished in Section S4 for the average daily use of E_2_ and EE_2_ by their respective users of 1.86 mg/cap·d and 21.7 µg/cap·d, suggests that a user of E_2_-OC can be approximated as being clinically equivalent to the average EE_2_-OC user. Under the *Switch to E_2_-OC* scenario, the expected load of steroidal estrogens that can be attributed to contraceptive choices made by *P_s_* can be modeled as follows:

$$\text{E}_{\text{E2-OC}}= \text{P}_{\text{s}}\cdot\text{[J}_{\text{d,E2 OC}}+\text{J}_{\text{f,E2 OC}}+\text{J}_{\text{L,E2 OC}}\text{]}$$

(S8)

*J_d,E2OC_*, *J_f,E2OC_* and *J_L,E2OC_* were estimated using Eq. (S1), (S2) and (S3), respectively. All other variables are as previously stated.
