# Supplementary material for: Contraceptive Options and Their Associated Estrogenic Environmental Loads: Relationships and Trade-Offs
Source: PLoS One. 2014 Mar 26;9(3):e92630. doi: 10.1371/journal.pone.0092630 (PMC3966801; doi:10.1371/journal.pone.0092630)
Supplement: File S9 — Nomenclature and Specific Estimates for All Variables Used. (DOC) [file pone.0092630.s009.doc]

# S9 Nomenclature and Specific Estimates for All Variables Used

**Table S7** Nomenclature and Specific Estimates for All Variables.

| **Parameter** | **Description** | **Estimate** | **Units** | | | **Data Source** | | | **Notes** |
| --- | --- | --- | --- | --- | --- | --- | --- | --- | --- |
|  |  |  | | |  |  | | |  |
| **A_Copper-T IUD_** | Annual probability that a typical user of Copper-T IUD will experience an unintended pregnancy during the first year of use due to contraception failure. | 0.8 | | | % | Trussel^l51^ | | |  |
| **A_Depo-Provera_** | Annual probability that a typical user of Depo-Provera will experience an unintended pregnancy during the first year of use due to contraception failure. | 6 | | | % | Trussel^l51^ | | |  |
| **A_Diaphragm_** | Annual probability that a typical user of diaphragms will experience an unintended pregnancy during the first year of use due to contraception failure. | 12 | | | % | Trussel^l51^ | | |  |
| **A_Female condom_** | Annual probability that a typical user of female condoms will experience an unintended pregnancy during the first year of use due to contraception failure. | 21 | | | % | Trussel^l51^ | | |  |
| **A_Fertility awareness-based methods_** | Annual probability that a typical user of fertility awareness-based methods will experience an unintended pregnancy during the first year of use due to contraception failure. | 24 | | | % | Trussel^l51^ | | |  |
| **A_Implanon_** | Annual probability that a typical user of Implanon will experience an unintended pregnancy during the first year of use due to contraception failure. | 0.05 | | | % | Trussel^l51^ | | |  |
| **A_LNG-20 IUS_** | Annual probability that a typical user of LNG-20 IUS will experience an unintended pregnancy during the first year of use due to contraception failure. | 0.2 | | | % | Trussel^l51^ | | |  |
| **A_Male condom_** | Annual probability that a typical user of male condoms will experience an unintended pregnancy during the first year of use due to contraception failure. | 18 | | | % | Trussel^l51^ | | |  |
| **A_Male sterilization_** | Annual probability that a typical user of male sterilization will experience an unintended pregnancy during the first year of use due to contraception failure. | 0.15 | | | % | Trussel^l51^ | | |  |
| **A_Nomethod_** | Annual probability of experiencing an unintended pregnancy among those who use no method of contraception. | 85 | | | % | Trussel^l51^ | | |  |
| **A_EE2-OC_** | Annual probability that a typical user of OC will experience an unintended pregnancy during the first year of use due to contraception failure. | 9 | | | % | Trussel^l51^ | | |  |
| **A_Progestin-only pill_** | Annual probability that a typical user of Progestin-only pills will experience an unintended pregnancy during the first year of use due to contraception failure. | 9 | | | % | Trussel^l51^ | | |  |
| **A_Spermicides_** | Annual probability that a typical user of spermicides will experience an unintended pregnancy during the first year of use due to contraception failure. | 28 | | | % | Trussel^l51^ | | |  |
| **A_Sponge-Nulliparous women_** | Annual probability that a typical nulliparous user of sponges will experience an unintended pregnancy during the first year of use due to contraception failure. | 12 | | | % | Trussel^l51^ | | |  |
| **A_Sponge-Parous women_** | Annual probability that a typical parous user of sponges will experience an unintended pregnancy during the first year of use due to contraception failure. | 24 | | | % | Trussel^l51^ | | |  |
| **A_Tubal ligation (Female sterilization)_** | Annual probability that a typical user of female sterilization will experience an unintended pregnancy during the first year of use due to contraception failure. | 0.5 | | | % | Trussel^l51^ | | |  |
| **A_Withdrawal_** | Annual probability that a typical user of the withdrawal method will experience an unintended pregnancy during the first year of use due to contraception failure. | 22 | | | % | Trussel^l51^ | | |  |
| **E_a_** | Total estrogenic load averted through a population’s collective use of contraception. | 4.80 | | | tonnes of E_2-eq_ | Eq. (S4) | | |  |
| **E_p_** | Estrogenic load that would have been released over the course of all pregnancies averted due to American’s collective use of contraception. | 0.66 | | | tonnes of E_2-eq_ | Eq. (S4a) | | |  |
| **E_L_** | Estrogenic load represented by all unwanted legacies averted through American’s use of contraception. | 4.44 | | | tonnes of E_2-eq_ | Eq. (S4b) | | |  |
| **E_e_** | Total estrogenic content directly released due to the current contraceptive mix used by Americans. | 0.31 | | | tonnes of E_2-eq_ | Section S1 | | |  |
| **E_EE2-OC_** | Total estrogenic load that can be associated with 1,000 first year users of EE_2_-OC | 62 | | | grams of E_2-eq_ | Eq. (S6) | | |  |
| **E_s_** | Total estrogenic load that can be associated with those who discontinue the use of EE_2_OC | 107 | | | grams of E_2-eq_ | Eq. (S7) | | |  |
| **E_E2-OC_** | Total estrogenic load that can be associated with 1,000 first year users of E_2_-OC | 137 | | | grams of E_2-eq_ | Eq. (S8) | | |  |
| **E_CIUD_** | Total estrogenic load that can be associated with 1,000 first year users of Copper IUDs | 2.8 | | | grams of E_2-eq_ | Eq. (S7) | | |  |
| **E_MC_** | Total estrogenic load that can be associated with 1,000 first year users of Male Condoms | 65 | | | grams of E_2-eq_ | Eq. (S7) | | |  |
| **E_Di_** | Total estrogenic load that can be associated with 1,000 first year users of Diaphragms | 43 | | | grams of E_2-eq_ | Eq. (S7) | | |  |
| **f_s_** | Fraction of wastewater that currently undergoes secondary treatment in the USA. | 0.47 | | |  | US EPA^36^ | | |  |
| **f_ad_** | Fraction of wastewater that currently undergoes advanced treatment in the USA. | 0.53 | | |  | US EPA^36^ | | |  |
| **f_a_** | Fraction of all pregnancies averted that would have resulted in the outcome of induced abortion. | 0.42 | | |  | Ventura et al.^7^; Hoover et al.^40^ | | |  |
| **f_b_** | Fraction of all pregnancies averted that would have resulted in the outcome of birth. | 0.44 | | |  | Ventura et al.^7^; Hoover et al.^40^ | | |  |
| **f_e_** | Fraction of all pregnancies averted that would have resulted in the outcome of ectopic pregnancy. | 0.006 | | |  | Ventura et al.^7^; Hoover et al.^40^ | | |  |
| **f_m_** | Fraction of all pregnancies averted that would have resulted in the outcome of spontaneous abortion. | 0.134 | | |  | Ventura et al.^7^; Hoover et al.^40^ | | |  |
| **f_a, all other methods_** | Fraction of unintended pregnancies experienced by the users of all other methods that will result in the outcome of induced abortion. | 0.46 | | |  | Trussell et al.^52^ | | |  |
| **f_a,Copper-T IUD_** | Fraction of unintended pregnancies experienced by the users of Copper-T IUD that will result in the outcome of induced abortion. | 0.45 | | |  | Trussell et al.^52^ | | |  |
| **f_a,LNG-20 IUS_** | Fraction of unintended pregnancies experienced by the users of LNG-20 IUS that will result in the outcome of induced abortion. | 0.23 | | |  | Trussell et al.^52^ | | |  |
| **f_a,Tubal ligation_** | Fraction of unintended pregnancies experienced by the users of tubal ligation that will result in the outcome of induced abortion. | 0.31 | | |  | Trussell et al.^52^ | | |  |
|  |  |  | | |  |  | | |  |
| **f_b, all other methods_** | Fraction of unintended pregnancies experienced by the users of all other methods that will result in the outcome of birth. | 0.37 | | |  | Trussell et al.^52^ | | |  |
| **f_b,Copper-T IUD_** | Fraction of unintended pregnancies experienced by the users of Copper-T IUD that will result in the outcome of birth. | 0.36 | | |  | Trussell et al.^52^ | | |  |
| **f_b,LNG-20 IUS_** | Fraction of unintended pregnancies experienced by the users of LNG-20 IUS that will result in the outcome of birth. | 0.19 | | |  | Trussell et al.^52^ | | |  |
| **f_b,Tubal ligation_** | Fraction of unintended pregnancies experienced by the users of tubal ligation that will result in the outcome of birth. | 0.25 | | |  | Trussell et al.^52^ | | |  |
|  |  |  | | |  |  | | |  |
| **f_e, all other methods_** | Fraction of unintended pregnancies experienced by the users of all other methods that will result in the outcome of ectopic pregnancy. | 0.01 | | |  | Trussell et al.^52^ | | |  |
| **f_e,Copper-T IUD_** | Fraction of unintended pregnancies experienced by the users of Copper-T IUD that will result in the outcome of ectopic pregnancy. | 0.03 | | |  | Trussell et al.^52^ | | |  |
| **f_e,LNG-20 IUS_** | Fraction of unintended pregnancies experienced by the users of LNG-20 IUS that will result in the outcome of ectopic pregnancy. | 0.50 | | |  | Trussell et al.^52^ | | |  |
| **f_e,Tubal ligation_** | Fraction of unintended pregnancies experienced by the users of tubal ligation that will result in the outcome of ectopic pregnancy. | 0.33 | | |  | Trussell et al.^52^ | | |  |
| **F_E1_/ U_E1_** | Fecal to urinary ratio with which E_1_ is released by pregnant women. | 0.13 | | |  | Adlercreutz et al.^11^;  Martin et al. ^12^ | | |  |
| **F_E1,E2_/ F_E2,E2_/**  **F_E3,E2_** | Fraction of an administered dose of E_2_ that is excreted as E_1_/E_2_/E_3_ and its conjugates via the user’s fecal excretions. | Minor | | |  | Assumed | | |  |
| **F_E2_/ U_E2_** | Fecal to urinary ratio with which E_2_ is released by pregnant women. | 0.84 | | |  | Adlercreutz et al.^11^;  Martin et al. ^12^ | | |  |
| **F_E3_/ U_E3_** | Fecal to urinary ratio with which E_3_ is released by pregnant women. | 0.03 | | |  | Adlercreutz et al.^11^;  Martin et al. ^12^ | | |  |
| **F_EE2,EE2_** | Fraction of an administered dose of EE_2_ that is excreted unchanged and its conjugates via the user’s fecal excretions. | 0.229 ± 0.03 | | |  | Section S3 | | | Assumed to be normally distributed. |
|  |  |  | | |  |  | | |  |
| **f_m, all other methods_** | Fraction of unintended pregnancies experienced by the users of all other methods that will result in the outcome of spontaneous abortion. | 0.17 | | |  | Trussell et al.^52^ | | |  |
| **f_m,Copper-T IUD_** | Fraction of unintended pregnancies experienced by the users of Copper-T IUD that will result in the outcome of spontaneous abortion. | 0.16 | | |  | Trussell et al.^52^ | | |  |
| **f_m,LNG-20 IUS_** | Fraction of unintended pregnancies experienced by the users of LNG-20 IUS that will result in the outcome of spontaneous abortion. | 0.09 | | |  | Trussell et al.^52^ | | |  |
| **f_m,Tubal ligation_** | Fraction of unintended pregnancies experienced by the users of tubal ligation that will result in the outcome of spontaneous abortion. | 0.11 | | |  | Trussell et al.^52^ | | |  |
| **f_no-method_** | Fraction of Ps that completely abandons the use of contraception after discounting the use of the EE_2_-OC. | 0.19 | | |  | Rosenberg  and Berg^45^ | | |  |
| **f_s,m_** | Fraction with which P_s_ adopts each option *m* (see table at right) after discontinuing the use  of EE_2_-OC. | | **m** | **f_s,m_** | | --- | --- | | Male Condom | 0.46 | | Rhythm, Withdrawal, or Natural Family Planning | 0.17  (This fraction was disaggregated among each option using the data of Mosher and Jones^5^) | | Sterilization | 0.06 | | IUD, Implant, Injectable (DMPA) | 0.05  (This fraction was disaggregated among each option using the data of Mosher and Jones^5^) | | Other Barrier or Spermicide | 0.05 | | Other | 0.03 | | | |  | Rosenberg  and Berg^45^ | | |  |
| **f_u_** | Fraction of unintended births that is unwanted. | 0.40 | | |  | Trussell et al.^52^  Trussell^53-54^ | |  | |
| **G_L_** | Duration of single birth’s genetic legacy in the USA. | 470 | | | years | Murtaugh and Schlax^55^ |  | | |
| **IP_0_** | The rate at which *P_T_* currently experiences unintended pregnancies. | 39 | | | Unintended pregnancies per 1000 women | Finer and Henshaw^47^ |  | | |
| **IP_e_** | The unintended pregnancy rate expected to be observed among the user population *P_T_* should they all stop using their respective contraceptive methods. | 354 | | | Unintended pregnancies per 1000 women | Trussell et al.^53^ |  | | |
| **J_d,EE2-OC_** | Steroidal estrogen load released directly via the use of EE_2_-OC. | 29.5 | | | mg of E_2-eq_/  user. first year of use | Eq. (S1) |  | | |
| **J_d,E2-OC_** | Steroidal estrogen load released directly via the use of E_2_-OC. | 105 | | | mg of E_2-eq_/  user. first year of use | Eq. (S1) |  | | |
| **J_f,no method_** | Load of natural estrogens released over the course of unintended pregnancies that can be associated with user’s choice to use no method of contraception. | 43 | | | mg of E_2-eq_/  user. first year of use | Eq. (S2) |  | | |
| **J_f,spermicide_** | Load of natural estrogens released over the course of unintended pregnancies that can be associated with user’s choice to use spermicides. | 14 | | | mg of E_2-eq_/  user. first year of use | Eq. (S2) |  | | |
| **J_f,fertility awareness-based methods_** | Load of natural estrogens released over the course of unintended pregnancies that can be associated with user’s choice to use fertility awareness-based methods. | 12 | | | mg of E_2-eq_/  user. first year of use | Eq. (S2) |  | | |
| **J_f,withdrawal_** | Load of natural estrogens released over the course of unintended pregnancies that can be associated with user’s choice to use withdrawal. | 11 | | | mg of E_2-eq_/  user. first year of use | Eq. (S2) |  | | |
| **J_f,sponge-parous_** | Load of natural estrogens released over the course of unintended pregnancies that can be associated with a parous user’s choice to use sponges. | 12 | | | mg of E_2-eq_/  user. first year of use | Eq. (S2) |  | | |
| **J_f,sponge-nulliparous_** | Load of natural estrogens released over the course of unintended pregnancies that can be associated with a nulliparous user’s choice to use sponges. | 6 | | | mg of E_2-eq_/  user. first year of use | Eq. (S2) |  | | |
| **J_f,female condom_** | Load of natural estrogens released over the course of unintended pregnancies that can be associated with a user’s choice to use female condoms. | 11 | | | mg of E_2-eq_/  user. first year of use | Eq. (S2) |  | | |
| **J_f,male condom_** | Load of natural estrogens released over the course of unintended pregnancies that can be associated with a user’s choice to use male condoms. | 9 | | | mg of E_2-eq_/  user. first year of use | Eq. (S2) |  | | |
| **J_f,diaphragm_** | Load of natural estrogens released over the course of unintended pregnancies that can be associated with a user’s choice to use diaphragms. | 6 | | | mg of E_2-eq_/  user. first year of use | Eq. (S2) |  | | |
| **J_f,EE2-OC_** | Load of natural estrogens released over the course of unintended pregnancies that can be associated with a user’s choice to use EE2-OC. | 5 | | | mg of E_2-eq_/  user. first year of use | Eq. (S2) |  | | |
| **J_f,E2-OC_** | Load of natural estrogens released over the course of unintended pregnancies that can be associated with a user’s choice to use E2-OC. | 5 | | | mg of E_2-eq_/  user. first year of use | Eq. (S2) |  | | |
| **J_f,progestin-only pill_** | Load of natural estrogens released over the course of unintended pregnancies that can be associated with a user’s choice to use progestin-only pill. | 5 | | | mg of E_2-eq_/  user. first year of use | Eq. (S2) |  | | |
| **J_f,depo-provera_** | Load of natural estrogens released over the course of unintended pregnancies that can be associated with a user’s choice to use Depo-provera. | 3 | | | mg of E_2-eq_/  user. first year of use | Eq. (S2) |  | | |
| **J_f,copper IUD_** | Load of natural estrogens released over the course of unintended pregnancies that can be associated with a user’s choice to use Copper IUD. | 0.4 | | | mg of E_2-eq_/  user. first year of use | Eq. (S2) |  | | |
| **J_f, IUS_** | Load of natural estrogens released over the course of unintended pregnancies that can be associated with a user’s choice to use Levengesterol IUS. | 0.1 | | | mg of E_2-eq_/  user. first year of use | Eq. (S2) |  | | |
| **J_f_,** _female sterilization_ | Load of natural estrogens released over the course of unintended pregnancies that can be associated with a user’s choice to use female sterilization. | 0.2 | | | mg of E_2-eq_/  user. first year of use | Eq. (S2) |  | | |
| **J_f_,** _male sterilization_ | Load of natural estrogens released over the course of unintended pregnancies that can be associated with a user’s choice to use male sterilization. | 0.1 | | | mg of E_2-eq_/  user. first year of use | Eq. (S2) |  | | |
| **J_f_,** _implant_ | Load of natural estrogens released over the course of unintended pregnancies that can be associated with a user’s choice to use the implant. | 0.03 | | | mg of E_2-eq_/  user. first year of use | Eq. (S2) |  | | |
| **J_L,no method_** | Legacy estrogenic load for the users of no method of contraception | 261 | | | mg of E_2-eq_/  user. first year of use | Eq. (S3) |  | | |
| **J_L,spermicide_** | Legacy estrogenic load for the users of spermicide | 87 | | | mg of E_2-eq_/  user. first year of use | Eq. (S3) |  | | |
| **J_L,fertility awareness-based methods_** | Legacy estrogenic load for the users of fertility awareness-based methods. | 74 | | | mg of E_2-eq_/  user. first year of use | Eq. (S3) |  | | |
| **J_L,withdrawal_** | Legacy estrogenic load for the users of withdrawal. | 68 | | | mg of E_2-eq_/  user. first year of use | Eq. (S3) |  | | |
| **J_L,sponge-parous_** | Legacy estrogenic load for the parous users of sponges. | 74 | | | mg of E_2-eq_/  user. first year of use | Eq. (S3) |  | | |
| **J_L,sponge-nulliparous_** | Legacy estrogenic load for the nulliparous users of sponges. | 37 | | | mg of E_2-eq_/  user. first year of use | Eq. (S3) |  | | |
| **J_L,female condom_** | Legacy estrogenic load for the users of female condoms. | 64 | | | mg of E_2-eq_/  user.first year of use | Eq. (S3) |  | | |
| **J_L,male condom_** | Legacy estrogenic load for the users of male condoms . | 56 | | | mg of E_2-eq_/  user.first year of use | Eq. (S3) |  | | |
| **J_L,diaphragm_** | Legacy estrogenic load for the users of diaphragms. | 37 | | | mg of E_2-eq_/  user. first year of use | Eq. (S3) |  | | |
| **J_L,EE2-OC_** | Legacy estrogenic load for the users of EE2-OC. | 28 | | | mg of E_2-eq_/  user. first year of use | Eq. (S3) |  | | |
| **J_L,E2-OC_** | Legacy estrogenic load for the users of E2-OC. | 28 | | | mg of E_2-eq_/  user. first year of use | Eq. (S3) |  | | |
| **J_L,progestin-only pill_** | Legacy estrogenic load for the users of progestin-only pill. | 28 | | | mg of E_2-eq_/  user.first year of use | Eq. (S3) |  | | |
| **J_L,depo-provera_** | Legacy estrogenic load for the users of Depo provera. | 19 | | | mg of E_2-eq_/  user.first year of use | Eq. (S3) |  | | |
| **J_L, copper IUD_** | Legacy estrogenic load for the users of Copper IUD. | 2.4 | | | mg of E_2-eq_/  user. first year of use | Eq. (S3) |  | | |
| **J_L, IUS_** | Legacy estrogenic load for the users of IUS. | 0.3 | | | mg of E_2-eq_/  user.first year of use | Eq. (S3) |  | | |
| **J_L, female sterilization_** | Legacy estrogenic load for the users of female sterilization. | 1.0 | | | mg of E_2-eq_/  user.first year of use | Eq. (S3) |  | | |
| **J_L, male sterilization_** | Legacy estrogenic load for the users of male sterilization. | 0.5 | | | mg of E_2-eq_/  user.first year of use | Eq. (S3) |  | | |
| **J_L, implant_** | Legacy estrogenic load of implant users. | 0.15 | | | mg of E_2-eq_/  user.first year of use | Eq. (S3) |  | | |
| **k-E1** | First order decay constant for E1 in surface waters. | 0.3 | | | d^-1^ | Caldwell et al.^17^ |  | | |
| **k-E2** | First order decay constant for E2 in surface waters. | 0.3 | | | d^-1^ | Caldwell et al.^17^ |  | | |
| **k-E3** | First order decay constant for E3 in surface waters. | 5.7 | | | d^-1^ | Caldwell et al.^17^ |  | | |
| **k-EE2** | First order decay constant for EE2 in surface waters. | 0.07 | | | d^-1^ | Caldwell et al.^17^ |  | | |
| **L_BF_** | Life-span averaged annual load of natural estrogens basally excreted by females. | 1.54 | | | mg of E_2-eq_  per year | Section S5 |  | | |
| **L_BM_** | Life-span averaged annual load of natural estrogens basally excreted by males. | 1.05 | | | mg of E_2-eq_  per year | Section S5 |  | | |
| **L_E2-OC_** | Per user daily consumption of E_2_ via users of OC. | 1419 | | | µg of E_2_  /user·d | Section S3 |  | | |
| **L_EE2-OC_** | Per user daily consumption of EE_2_ via users of OC. | 21.7 | | | µg of EE_2_  /user·d | Section S3 |  | | |
| **L_FT_** | Life-span averaged annual load of natural estrogens due to an assumed total fertility rate of 1.85. | 6.5 | | | mg of E_2-eq_  per year | Section S5 |  | | |
| **M_L_** | Estrogenic legacy load of unwanted births and its genetic lineage | 2.1 | | | g of E_2-eq_  per unwanted birth | Eq. (S3a) |  | | |
| **Mw_E1_** | Molecular weight of E_1_. | 270.4 | | | g/mole |  |  | | |
| **Mw_E2_** | Molecular weight of E_2_. | 272.4 | | | g/mole |  |  | | |
| **Mw_E3_** | Molecular weight of E_3_. | 288.4 | | | g/mole |  |  | | |
| **Mw_EE2_** | Molecular weight of EE_2_. | 296.4 | | | g/mole |  |  | | |
| **N_averted_** | Net number of pregnancy events averted through a population’s collective use of contraception. | 8.8 | | | Million people | Eq. (S5) |  | | |
| **N_a-averted_** | Net number of abortions averted through a population’s collective use of contraception. | 5.0 | | | Million people | Eq. (S5a) |  | | |
| **N_ub-averted_** | Net number of unwanted births averted through a population’s collective use of contraception. | 2.1 | | | Million people | Eq. (S5b) |  | | |
|  |  |  | | |  |  |  | | |
| **N_m-averted_** | Net number of miscarriages averted through a population’s collective use of contraception. | 1.6 | | | Million people | Eq. (S5c) |  | | |
| **N_e-averted_** | Net number of ectopic pregnancies averted through a population’s collective use of contraception. | 0.07 | | | Million people | Eq. (S5d) |  | | |
| **P_E1_** | Potency of E_1_ relative to E_2_. | 1/3 | | |  | Section S2 |  | | |
| **P_E2_** | Potency of E_2_ relative to E_2_. | 1 | | |  | By definition Section S2 |  | | |
| **P_E3_** | Potency of E_3_ relative to E_2_. | 1/25 | | |  | Section S2 |  | | |
| **P_EE2_** | Potency of EE_2_ relative to E_2_. | 10 | | |  | Section S2 |  | | |
| **P_s_** | Unit population used a basis for scenario analysis. | 1,000 | | | First-year users of contraception | Assumed |  | | |
| **P_T_** | Total number of American women using any form of contraception. | 38,106,259 | | | People | Mosher and Jones^5^ |  | | |
| **R_s,E1_/R_s,E2_**  **/R_s,E3_/R_s,EE2_** | Removal of steroidal estrogens in secondary treatment plants. | 66.8% (**R_s,E1_**), 85.0% (**R_s,E2_**), 97.0% (**R_s,E3_**), 84.0% (**R_s,EE2_**) | | |  | Caldwell et al.^17^ |  | | |
| **R_ad,E1_/R_ad,E2_**  **/R_ad,E3_/R_ad,EE2_** | Removal of steroidal estrogens in advanced treatment plants. | 87.7% (**R_s,E1_**), 95.5% (**R_s,E2_**), 97.0% (**R_s,E3_**), 84.0% (**R_s,EE2_**) | | |  | Caldwell et al.^17^ |  | | |
| **SR_F_** | Fraction of births in the USA that are females. | 0.49 | | |  | Martin et al.^56^ |  | | |
| **SR_M_** | Fraction of births in the US that are males. | 0.51 | |  | | Martin et al.^56^ |  | | |
| **t_a_** | Duration of an unintended pregnancy resulting in the outcome of induced abortion. | | **Weeks** | **Days** | **Cumulative Probability (%)** | | --- | --- | --- | | 0 | 0 | 0 | | 4 | 28 | 31 | | 7 | 49 | 49 | | 8 | 56 | 63 | | 9 | 63 | 72 | | 10 | 70 | 79 | | 11 | 77 | 84 | | 12 | 84 | 88 | | 13 | 91 | 91 | | 14.5 | 101.5 | 95 | | 16.5 | 115.5 | 97 | | 19 | 133 | 99 | | 24 | 168 | 100 | | | | Weeks/Days | Pazol et al.^13^ |  | | |
| **t_b_** | Duration of an unintended pregnancy resulting in the outcome of birth. | | **Weeks** | **Days** | **Cumulative Probability (%)** | | --- | --- | --- | | 0 | 0 | 0 | | 30 | 210 | 1 | | 33.5 | 234.5 | 2 | | 35 | 245 | 3 | | 36 | 252 | 6 | | 37 | 259 | 10 | | 38 | 266 | 21 | | 39 | 273 | 43 | | 40 | 280 | 75 | | 41 | 287 | 90 | | 42 | 294 | 98 | | 43 | 301 | 100 | | 44 | 308 | 100 | | | | Weeks/Days | Goldhaber and Fireman^41^ |  | | |
| **t_e_** | Duration of an unintended pregnancy resulting in the outcome of ectopic pregnancy. | | **Weeks** | **Days** | **Cumulative Probability (%)** | | --- | --- | --- | | 0 | 0 | 0 | | 5 | 35 | 4 | | 6 | 42 | 7 | | 7 | 49 | 22 | | 8 | 56 | 37 | | 9 | 63 | 56 | | 10 | 70 | 67 | | 11 | 77 | 81 | | 12 | 84 | 85 | | 14 | 98 | 89 | | 17 | 119 | 93 | | 18 | 126 | 100 | | | | Weeks/Days | Goldhaber and Fireman^41^ |  | | |
| **t_m_** | Duration of an unintended pregnancy resulting in the outcome of spontaneous abortion. | | **Weeks** | **Days** | **Cumulative Probability (%)** | | --- | --- | --- | | 0 | 0 | 0 | | 4 | 28 | 14 | | 6.5 | 45.5 | 46 | | 10.5 | 73.5 | 75 | | 14.5 | 101.5 | 86 | | 18.5 | 129.5 | 95 | | 24 | 168 | 100 | | | | Weeks/Days | Jones and Kost^14^ |  | | |
| **t_r_** | Typical residence time of a wastewater parcel in surface water. | 40 | | | Days | Sinclair et al.^37^ |  | | |
| ∫**U_E1_** | Excretion of E_1_ as a function of gestational age | Was modelled by fitting appropriate distribution in @RISK to excretion developed from the data of Berg and Kuss^8^ and  Knuppel et al.^9^:  **Birth:** RiskBetaGeneral(3.4,17.7,18.5,1405)  **Abortions:** RiskLognorm(11.3,5.3)  **Miscarriage:** RiskBetaGeneral(1.36,4.2,6.3,54.8)  **Ectopic:** RiskBetaGeneral(1.24,3.8,6.1,40.3) | | | mg of E_1_ |  |  | | |
| **U_E1,E2_** | Fraction of an administered dose of E_2_ that is excreted as E_1_ and its conjugates via the user’s urinary excretions. | 0.30 ± 0.09 | | |  | Friel et al.^15^ | Assumed to be normally distributed. | | |
| ∫**U_E2_** | Excretion of E_2_ as a function of gestational age | Was modelled by fitting appropriate distributionin @RISK to excretion developed from the data of Berg and Kuss^8^ and  Knuppel et al.^9^:  **Birth:** RiskTriang(29.61,51.5,149.7)  **Abortions:** RiskLognorm(1.91,2.16,RiskShift(0.75))  **Miscarriage:** RiskLognorm(3.28,3.11,RiskShift(1.12))  **Ectopic:** RiskLognorm(2.56,2.89,RiskShift(0.95)) | | | mg of E_2_ | Berg and Kuss^8^; Knuppel et al.^9^ |  | | |
| **U_E2,E2_** | Fraction of an administered dose of E_2_ that is excreted unchanged and its conjugates via the user’s urinary excretions. | 0.10 ± 0.04 | | |  | Friel et al.^15^ | Assumed to be normally distributed. | | |
| ∫**U_E3_** | Excretion of E_3_ as a function of gestational age | Was modelled by fitting appropriate distributio in @RISK to excretion developed from the data of Berg and Kuss^8^ and  Knuppel et al.^9^:  **Birth:** RiskTriang(345.9,12,3020)  **Abortions:** RiskWeibull(1.83,26.3)  **Miscarriage:** RiskLognorm(101,25.7,RiskShift(-54.2))  **Ectopic:** RiskLognorm(60.3,19.2,RiskShift(-29.9)) | | | mg of E_3_ | Berg and Kuss^8^; Knuppel et al.^9^ |  | | |
| **U_E3,E2_** | Fraction of an administered dose of E_2_ that is excreted as E_3_ and its conjugates via the user’s urinary excretions. | 0.06 ± 0.04 | | |  | Friel et al.^15^ | Assumed to be normally distributed. | | |
| **U_EE2,EE2_** | Fraction of an administered dose of EE_2_ that is excreted unchanged and its conjugates via the user’s urinary excretions. | 0.143 ± 0.03 | | |  | Section S3 | Assumed to be normally distributed. | | |
