# Supplementary material for: Contraceptive Options and Their Associated Estrogenic Environmental Loads: Relationships and Trade-Offs
Source: PLoS One. 2014 Mar 26;9(3):e92630. doi: 10.1371/journal.pone.0092630 (PMC3966801; doi:10.1371/journal.pone.0092630)
Supplement: File S11 — Changes in Flows of Estrogens When Users of EE2-OC Switch to Diaphragms. (DOC) [file pone.0092630.s011.doc]

# S11 Changes in Flows of Estrogens When Users of EE_2_-OC Switch to Diaphragms

**4.6**

**(2.0,5.2)**

**E_2-eq_**

**29.5**

**(27,32)**

**EE_2_**

**Status quo Scenario**

**P_s_**

1, 000

**Pre-Treatment Associative Loads**

**EE_2_ used**

7.9 g/yr

**
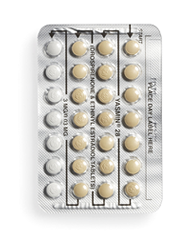
**


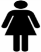

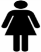

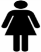

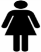

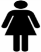

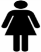

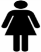

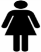

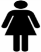

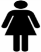

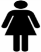

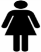


21212121

**Metabolic**

**Loss**

21212121

**E_2-eq_**

**EE_2_**

**29.5**

**(27,32)**

**2.95**

**(2.7, 3.2)**

#
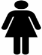

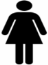

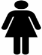


**Failures**


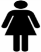

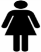

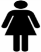

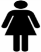

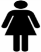

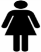

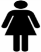

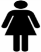

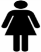

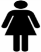

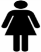

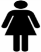


**Ectopic**

**1.2**

**Abortions**

**54.6**

**Fetal Losses**

**20.2**

**Mistimed Births**

**26.4**

**Unwanted Births**

**17.6**

**Unwanted Legacy**

**17.6**

21212121

**E_2-eq_**

**37.0**

**Diaphragm**

21212121

21212121

21212121

21212121

**Unwanted Legacy**

**13.2**

**Failures**

21212121

21212121

**Ectopic**

**0.9**

**Abortions**

**41.0**

**Fetal Losses**

**15.2**

**Mistimed Births**

**19.8**

**Unwanted Births**

**13.2**

21212121

**EE_2_**

**21**

(22,24)

**Legend**

**Figure S2: Changes in** **associative loads of steroidal estrogens when a unit of population of 1,000 first-year EE_2_-OC users switches entirely to the use of diaphragms.** *E_EE2-OC_* was estimated using Eq. (S6), while *E_Di_* was estimated using the appropriately modified version of Eq. (S7).

**P_s_**

1, 000

**Contraceptive Profile Adopted**

**100% Diaphragms**

**Unintended**

**Pregnancies**

120

**E_Di_**

**E_2-eq_**

**43**

**E_1_**

**6.0**

**(4.3, 7.3)**

(22,24)

**E_2_**

**2.9**

**(2.2, 3.5)**

**E_3_**

**30**

**(23, 37)**

**E_2-eq_**

**6.1**

**(5.2, 6.9)**

**E_2-eq_**

**62**

**E_EE2-OC_**

**Unintended**

**Pregnancies**

90

**E_3_**

**23**

**(17, 28)**

**E_2_**

**2.2**

**( 1.7, 2.7)**

**E_1_**

**4.5**

**( 3.5, 5.5)**

(22,24)

**E_2-eq_**

**4.6**

**(2.0,5.2)**

**E_2-eq_**

**27.7**

**Contraceptive Profile**

**100%** EE_2_-OC

**25%, 75%**

**Percentile**

**Expressed as**

**Grams/year**
