# Supplementary material for: Contraceptive Options and Their Associated Estrogenic Environmental Loads: Relationships and Trade-Offs
Source: PLoS One. 2014 Mar 26;9(3):e92630. doi: 10.1371/journal.pone.0092630 (PMC3966801; doi:10.1371/journal.pone.0092630)
Supplement: File S12 — Changes in Flows of Estrogens When Users of EE2-OC Switch to Copper IUDs. (DOC) [file pone.0092630.s012.doc]

# S12 Changes in Flows of Estrogens When Users of EE_2_-OC Switch to Copper IUDs

#
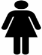

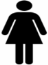

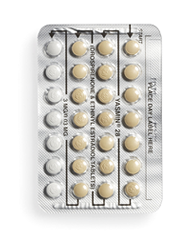


21212121

**Failures**


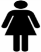

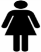

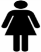

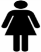

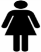

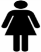

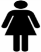

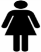

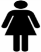

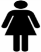

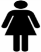

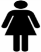


**Ectopic**

**0.2**

**Abortions**

**3.6**

**Fetal Losses**

**1.3**

**Mistimed Births**

**1.7**

**Unwanted Births**

**1.1**

**Unwanted Legacy**

**1.1**

**Copper IUD**

21212121

21212121

21212121

21212121

**EE_2_**

**21**

(22,24)

**Legend**

21212121

21212121

**Unwanted Legacy**

**13.2**

**Failures**

**Metabolic**

**Loss**


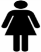

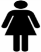

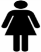

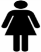

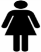

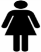

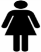

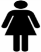

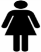

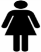

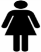

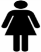


21212121

21212121

21212121

**Ectopic**

**0.9**

**Abortions**

**41.0**

**Fetal Losses**

**15.2**

**Mistimed Births**

**19.8**

**Unwanted Births**

**13.2**

**Status quo Scenario**

**Figure S3: Changes in** **associative loads of steroidal estrogens when a unit of population of 1,000 first-year EE_2_-OC users switches entirely to the use of Copper IUD.** *E_EE2-OC_* was estimated using Eq. (S6), while *E_CIUD_* was estimated using the appropriately modified version of Eq. (S7).

**E_2-eq_**

**62**

**E_EE2-OC_**

**P_s_**

1, 000

**Contraceptive Profile Adopted**

**100% Copper IUD**

**Unintended**

**Pregnancies**

8.0

**E_CIUD_**

**E_2-eq_**

**2.8**

**E_1_**

**0.40**

**(0.29,0.48)**

(22,24)

**E_2_**

**0.19**

**(0.15, 0.23)**

**E_3_**

**2.0**

**(1.5, 2.5)**

**E_2-eq_**

**0.40**

**(0.34,0.46)**

**25%, 75%**

**Percentile**

**Expressed as**

**Grams/year**

**E_2-eq_**

**2.4**

**Unintended**

**Pregnancies**

90

**E_3_**

**23**

**(17, 28)**

**E_2_**

**2.2**

**(1.7, 2.7)**

**E_1_**

**4.5**

**(3.5, 5.5)**

(22,24)

**E_2-eq_**

**4.6**

**(2.0,5.2)**

**P_s_**

1, 000

**E_2-eq_**

**29.5**

**(27,32)**

**Pre-Treatment Associative Loads**

**EE_2_**

**2.95**

**(2.7, 3.2)**

**E_2-eq_**

**27.7**

**Contraceptive Profile**

**100%** EE_2_-OC

**EE_2_ used**

7.9 g/yr
