# Supplementary material for: Contraceptive Options and Their Associated Estrogenic Environmental Loads: Relationships and Trade-Offs
Source: PLoS One. 2014 Mar 26;9(3):e92630. doi: 10.1371/journal.pone.0092630 (PMC3966801; doi:10.1371/journal.pone.0092630)
Supplement: File S13 — Flow of Estrogens Associated with the Use of E2-OC. (DOC) [file pone.0092630.s013.doc]

# S13 Flow of Estrogens Associated with the Use of E_2_
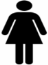

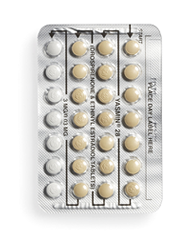
-OC

**Unwanted Legacy**

**13.2**

**Failures**

**Metabolic**

**Transformation**


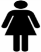

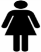

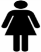

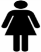

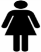

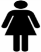

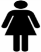

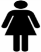

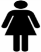

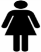

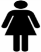

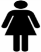


21212121

21212121

21212121

21212121

**E_2-eq_**

**27.7**

**Ectopic**

**0.9**

**Abortions**

**41.0**

**Fetal Losses**

**15.1**

**Mistimed Births**

**19.8**

**Unwanted Births**

**13.2**

**E_2_-OC Use**

21212121

21212121

**E_2-eq_**

**137**

**Figure S4: Loads of steroidal estrogens associated with a unit of population of 1000 first-year E_2_-OC users.** For further details on the estimation methodology used to model this scenario please refer to S8.

**Unintended**

**Pregnancies**

90.0

**E_3_**

**23**

**(17, 28)**

**E_2_**

**2.2**

**(1.7,2.7)**

**E_1_**

**4.5**

**(3.5,5.5)**

(22,24)

**E_2-eq_**

**4.6**

**(2.0, 5.2)**

**P_s_**

1, 000

**E_2-eq_**

**105**

**(88,123)**

**Pre-Treatment Associative Loads**

**Contraceptive Profile**

**100%** E_2_-OC

**E_2_ used**

518 g/yr

**E_3_**

**31**

**(17,45)**

**E_2_**

**52**

**(38,66)**

**E_1_**

**155**

**(124,187)**

(22,24)
