# Supplementary material for: Contraceptive Options and Their Associated Estrogenic Environmental Loads: Relationships and Trade-Offs
Source: PLoS One. 2014 Mar 26;9(3):e92630. doi: 10.1371/journal.pone.0092630 (PMC3966801; doi:10.1371/journal.pone.0092630)
Supplement: File S14 — Excretion of Equine Estrogens and their Relevance. (DOC) [file pone.0092630.s014.doc]

# S14 Excretion of Equine Estrogens and their Relevance

Even though the potential presence of equine estrogens in human urine has been suspected for more than half a century^34^, to our knowledge only urine from menopausal women has been analyzed for the endogenous presence of these compounds.^30,31^ Therefore, at present, it is not possible to estimate the release of these estrogens for population cohorts other than menopausal women.

In Table S8, the endogenous release of equine estrogens by menopausal women is estimated. In Table S9, the release of these estrogens from the exogenous use of conjugated and esterified estrogens is estimated where possible.

**Table S8** Endogenous release of equine estrogens by menopausal women.

| **Equine estrogen** | **Daily per capita excretion ^(a)^** | **Endogenous Load^(b)^** |
| --- | --- | --- |
|  | **µg/cap·d** | **Kg/yr** |
| 17αDihydroequilin | 1.2 | 19 |
| 17βDihydroequilin | 1.2 | 19 |
| 17αDihydroequilenin | 3.2 | 48 |
| 17βDihydroequilenin | Interference during analysis | ? |
| Equilin | 4.4 | 66 |
| Equilenin | < level of detection | ? |
|  |  |  |

(a) Sample weighted mean^30,31^; (b) Daily per capita excretion (µg/cap·d) × Number of women above the age of 51 years^2^ × 365 (d/yr) × 1/10^9^ (kg/µg).

**Table S9** Release of equine estrogens from the use of HRT preparations.

| **Equine estrogen** | **Conjugated Estrogen Metabolism^30^** | **Esterified Estrogen Metabolism** | | | | **Exogenous Load^(b)^** |
| --- | --- | --- | --- | --- | --- | --- |
|  | **g of Estrogen**  **/g of Conjugated Estrogen(%)** | | **g of Estrogen**  **/g of Esterified Estrogen (%)** | | **kg/yr** | |
| 17αDihydroequilin | 0.54 | | Not Available | | >3 | |
| 17βDihydroequilin | 0.86 | | 0.34^(a)^ | | 5 | |
| 17αDihydroequilenin | 1.57 | | Not Available | | >8 | |
| 17βDihydroequilenin | Interference | | Not Available | | ? | |
| Equilin | 0.34 | | 0.13^(a)^ | | 2 | |
| Equilenin | < level of detection | | Not Available | | ? | |
|  |  | |  |  | | |

(a) Mass fraction of conjugated estrogen excreted as the equine estrogen^30^ x [Average fraction of Esterified Estrogen (USP) that is equilin / Average fraction of Conjugated Estrogen (USP) that is equilin]; (b) [Annual consumption of conjugated estrogens in the USA^4^ x Conjugated estrogen metabolism] + [Annual consumption of Esterified Estrogens in the USA^4^ x Esterified estrogen metabolism]

In Table S10, the contribution of equine estrogens to the environment from menopausal women is estimated. A comparison of these results with those estimated in Table S1 for traditional estrogens suggests that equine estrogen from menopausal women alone could be significant contributors of estrogenic activity to the environment.

**Table S10** Release of equine estrogens by menopausal women.

| **Equine estrogen** | **Net Load Released to STPs by only menopausal women in the USA** | |
| --- | --- | --- |
|  | **kg of Equine Estrogen/yr ^(a)^** | **kg of E_2-eq_/yr ^(b)^** |
| 17αDihydroequilin | 22 | 0.6 |
| 17βDihydroequilin | 24 | 837.6 |
| 17αDihydroequilenin | 56 | 3.6 |
| 17βDihydroequilenin | ? | ? |
| Equilin | 68 | 17.4 |
| Equilenin | ? | ? |

(a) From Table S8 and S9; (b) Using in-vitro binding potency to the alpha estrogen receptor of Roach^35^.
