# Supplementary material for: Contraceptive Options and Their Associated Estrogenic Environmental Loads: Relationships and Trade-Offs
Source: PLoS One. 2014 Mar 26;9(3):e92630. doi: 10.1371/journal.pone.0092630 (PMC3966801; doi:10.1371/journal.pone.0092630)
Supplement: File S15 — References for All Supporting Information Sections. (DOC) [file pone.0092630.s015.doc]

# S15 References for All Supporting Information Sections

1. Chumlea WC, Schubert CM, Roche AF, Kulin HE, Lee PA, et al. (2003) Age at menarche and racial comparisons in US girls. Pediatrics 111: 110-113.
2. US Census (2011). IDB Database. US Census.
3. Shi L, Remer T, Buyken AE, Hartmann MF, Hoffmann P, et al. (2010) Prepubertal urinary estrogen excretion and its relationship with pubertal timing. American Journal of Physiology - Endocrinology and Metabolism 299: E990–E997.
4. Anderson PD, Johnson AC, Pfeiffer D, Caldwell DJ, Hannah R, et al. (2012) Endocrine disruption due to estrogens derived from humans predicted to be low in the majority of U.S. surface waters. Environmental Toxicology and Chemistry *31*: 1407–1415.
5. Mosher WD, Jones, J (2010) Use of contraception in the United States: 1982–2008. Vital Health Statistics (Series 23 Number 29): National Center for Health Statistics: Hyattsville, MD.
6. Gold EB, Bromberger J, Crawford S, Samuels S, Greendale GA, et al. (2001) Factors associated with age at natural menopause in a multiethnic sample of midlife women. American Journal of Epidemiology 153: 865-874.
7. Ventura SJ, Curtin SC, Abma JC, Henshaw SK (2010) Estimated pregnancy rates and rates of pregnancy outcomes for the United States, 1990-2008. National vital statistics reports (Vol 60 Number 7): National Center for Health Statistics: Hyattsville, MD.
8. Berg FD, Kuss E (1992) Serum concentration, urinary excretion of “classical” estrogens, catechol estrogens and 2-methoxyestrogens in normal human pregnancy. Archives of Gynecology and Obstetrics 251: 17–27.
9. Knuppel RA, Sbarra AJ, Cetrulo CL, Kappy KA, Ingardia CJ, et al. (1979) 24-hour urine creatinine excretion in pregnancy. Obstetrics & Gynecology 54: 327–329.
10. Wise AO, Brien K, Woodruff T (2011) Are oral contraceptives a significant contributor to the estrogenicity of drinking water? Environmental Science and Technology 45 :51–60.
11. Adlercreutz H, [Martin F](http://www.tandfonline.com/action/doSearch?action=runSearch&type=advanced&result=true&prevSearch=%2Bauthorsfield%3A(Martin%2C+F)), [Pulkkinen M](http://www.tandfonline.com/action/doSearch?action=runSearch&type=advanced&result=true&prevSearch=%2Bauthorsfield%3A(Pulkkinen%2C+M)), [Dencker H](http://www.tandfonline.com/action/doSearch?action=runSearch&type=advanced&result=true&prevSearch=%2Bauthorsfield%3A(Dencker%2C+H)), [Rimer U](http://www.tandfonline.com/action/doSearch?action=runSearch&type=advanced&result=true&prevSearch=%2Bauthorsfield%3A(Rimer%2C+U)), et al. (1976) Intestinal metabolism of estrogens*.*The Journal of Clinical Endocrinology & Metabolism 43: 497–505.

1. Martin F, Peltonen J, Laatikainen T, Pulkkinen M, Adlercreutz H (1975) Excretion of progesterone metabolites and estriol in faeces from pregnant women during ampicillin administration. Journal of Steroid Biochemistry 6: 1339–1346.
2. Pazol K, Gamble SB, Parker WY, Cook DA, Zane SB, et al. (2009) Abortion surveillance United States, 2006. Morbidity and Mortality Weekly Report -Surveillance Summaries 58: 1–35.
3. Jones RK, Kost K (2007) Underreporting of induced and spontaneous abortion in the United States: an analysis of the 2002 National Survey of Family Growth. Studies in Family Planning 38: 187–197.
4. Friel PN, Hinchcliffe C, Wright JV (2005) Hormone replacement with estradiol: Conventional oral doses result in excessive exposure to estrone. Alternative Medicine Review 10: 36–41.
5. Biro FM, Lucky AW, Huster GA, Morrison JA (1995) Pubertal staging in boys*.* Journal of Pediatrics 127: 100–102.
6. Caldwell DJ, Mastrocco F, Nowak E, Johnston J, Yekel H, et al. (2010) An assessment of potential exposure and risk from estrogens in drinking water. Environmental Health Perspective 118: 338–344.
7. Kamyab S, Fotherby K, Steele SJ (1969) Metabolism of 4-14C-ethynyl oestradiol in women. Nature 221: 360-361.
8. Abdel-Aziz MT, Williams KIH (1970) Metabolism of radioactive 17α-ethynylestradiol by women. Steroids 15: 695−710.
9. Reed MJ, Fotherby K, Steel SJ (1972) Metabolism of ethynyloestradiol in man. Journal of Endocrinology 55: 351-361.
10. Williams MC, Helton ED, Goldzieher JW (1975) Urinary metabolites of 17-alpha-ethynylestradiol-9-alpha,11-xi-h-3 in women—chromatographic profiling and identification of ethynyl and non-ethynyl compounds. Steroids 25: 229–246.
11. Helton ED, Williams MC, Goldzieher JW (1976) Human urinary and liver conjugates of 17α-ethynylestradiol. Steroids 27: 851–867.
12. Speck U, Wendt H, Schulze PE (1976) [Bio-availability and pharmacokinetics of cyproterone acetate-14C and ethinyloestradiol-3H after oral administration as a coated tablet.](http://www.ncbi.nlm.nih.gov/pubmed/949892) Contraception 14: 151-163.
13. Williams MC, Goldzieher JW (1980) Chromatographic patterns of urinary ethynyl estrogen metabolites in various populations. Steroids 36: 255-282.
14. Maggs JL, Grimmer SFM, L’ E-Orme M, Breckerridge AM, Park BK, et al. (1983) The biliary and urinary metabolites of [3H] 17a-ethynylestradiol in women. Xenobiotica 13 :421–431.
15. Back DJ, Maggs JL, Purba HS, Newby S, Park BK (1984) 2-hydroxylation of ethinyloestradiol in relation to the oxidation of sparteine and antipyrine. British Journal of Clinical Pharmacology 18: 603-607.
16. Maggs JL , Park BK (1985) A comparative study of biliary and urinary 2-hydroxylated metabolites of [6, 7–3H] 17′-ethynylestradiol in women. Contraception 32:173-182.
17. Johnson AC, Williams RJ (2004) A model to estimate influent and effluent concentrations of estradiol, estrone, and ethinylestradiol at sewage treatment works. Environmental Science & Technology 38: 3649– 3658.
18. Cargill DI, Steinetz BG, Gosnell E, Beach VL, Meli A, Fujimoto GI, et al. (1969) Fate of ingested radiolabelled ethynylestradiol and its 3-cyclopentyl ether in patients with bile fistulas. The Journal of Clinical Endocrinology & Metabolism 29: 1051–1061.
19. Adams WP, Hasegawa J, Johnson RN, Haring RC (1979) Conjugated estrogens bioinequivalence: comparison of four products in postmenopausal women. Journal of Pharmaceutical Sciences 68: 986–991.
20. Johnson RN, Masserano RP, Kho BT, Adams WP (1979) Steady-state urinary excretion method for determining bioequivalence of conjugated estrogen products. Journal of Pharmaceutical Sciences 67: 1218–1224.
21. Migeon CJ, Wall PE, Bertrand J (1959) Some aspects of the metabolism of estrone in normal individuals. Journal of Clinical Investigation 38: 619–629.
22. Levitz M, Spitzer JR, Twombly GH (1958) Interconversions of 16-oxygenated estrogens. I. The synthesis of estriol-16-C14 and its metabolism in man. Journal of Biological Chemistry 231: 787-797.
23. Stárka L, Breuer H. Cedard L (1966) Biosynthesis of equilin and related equine oestrogens in perfused human placenta. Journal of Endocrinology 34: 447–456.
24. Tyler CR, Filby AL, Bickley LK, Cumming RI, Gibson R, Labadie P, et al. (2009) Environmental health impacts of equine estrogens derived from hormone replacement therapy. Environmental Science and Technology 43: 3897–3904.
25. US EPA (2008). Clean Watersheds Needs Survey 2004 Report to Congress. US EPA.
26. Sinclair CJ, Boxall ABA, Parsons SA, Thomas MR (2006) Prioritization of pesticide environmental transformation products in drinking water supplies. Environmental Science & Technology 40: 7283–7289.
27. Hall KS, Trussell J (2012) Types of combined oral contraceptives used by US women. Contraception 86: 659-665.
28. Bayer (2012). Natazia Monograph. Bayer, Wayne, NJ.
29. Hoover KW, Tao G, Kent CK (2001) Trends in the diagnosis and treatment of ectopic pregnancy in the United States. Obstetrics & Gynecology 115: 495–502.
30. Goldhaber MK, Fireman BH (1991) The fetal life table revisited: spontaneous abortion rates in three Kaiser Permanente cohorts. Epidemiology 2: 33–39.
31. SCHER (2011) Opinion on Chemicals And The Water Framework Directive: Draft Environmental Quality Standards – Estradiol; European Commission (EU): Brussels.
32. SCHER (2011) Opinion on Chemicals And The Water Framework Directive: Draft Environmental Quality Standards – Ethinylestradiol; European Commission (EU): Brussels.
33. Caldwell DJ, Mastrocco F, Anderson PD, Länge R, Sumpter JP (2012) Predicted-no-effect concentrations for the steroid estrogens estrone, 17β-estradiol, estriol, and 17α-ethinyl estradiol. Environmental Toxicology and Chemistry 31: 1396– 1406.
34. Rosenberg MJ, Waugh MS (1998) Oral contraceptive discontinuation: a prospective evaluation of frequency and reasons. American Journal of Obstetrics & Gynecology 179, 577–582.
35. Williams RJ, Keller VDJ, Johnson AC, Young AR, Holmes MGR, et al. (2009) A national risk assessment for intersex in fish arising from steroid estrogens. Environmental Toxicology and Chemistry 28: 220-230.
36. Finer LB, Henshaw SK (2006) Disparities in rates of unintended pregnancy in the United States, 1994 and 2001*.* Perspectives on Sexual and Reproductive Health 38: 90–96.
37. FDA (2012). [Drugs@FDA](http://www.accessdata.fda.gov/scripts/cder/drugsatfda/index.cfm) -Database. FDA.
38. Miniño AM, Murphy SL, Xu JQ, Kochanek KD (2011) Deaths: Final data for 2008. National vital statistics reports; vol 59 no 10. Hyattsville, MD: National Center for Health Statistics.
39. U.N. Secretariat (2007) World population prospects, the 2006 revision. New York, The Department of Economic and Social Affairs, United Nations.
40. Trussell J (2011) Contraceptive failure in the United States. Contraception 81:397–404.
41. Trussell J, Lalla AM, Doan QV, Reyes E, Pinto L et al. (2009) Cost effectiveness of contraceptives in the United States. Contraception 79: 5-14.
42. Trussell J (2007) The cost of unintended pregnancy in the United States. Contraception 75(3): 168-170.
43. Trussell J (2008) Overstating the cost savings from contraceptive use. European Journal of Contraception and Reproductive Health Care 13(3): 219-221.
44. Murtaugh PA, Schlax MG (2009) Reproduction and the carbon legacies of individuals. Global Environmental Change 19(1): 14–20.
45. Martin JA, Hamilton BE, Sutton PD, Ventura SJ, Menacker F, Kirmeyer, S (2011) Births: final data for 2009. National Vital Statistics Reports 60: 1-72.
46. Yamamoto T (1969) Sex differentiation In *Fish Physiology;* Hoar, W.S., Rordall, D.J., Eds.; Academic Press: New York; pp. 117–175.
47. Yamamoto T (1965) Estriol-induced XY females of the medaka (*Oryzias latipes*) and their progenies. General and Comparative Endocrinology 5: 527–533.
48. U.N. Secretariat (2011) World population prospects, the 2010 revision. New York, The Department of Economic and Social Affairs, United Nations.
49. Alkema L, Raftery A, Gerland P, Clark S, Pelletier F, Buettner T, et al. (2011) Probabilistic projections of the total fertility rate for all countries. Demography 48 (3): 815-839.
50. Anonymous (2010) Natazia--a new oral contraceptive. The Medical Letter on Drugs and Therapeutics 52(1346): 71-2.
